# Resistin-like molecules: a marker, mediator and therapeutic target for multiple diseases

**DOI:** 10.1186/s12964-022-01032-w

**Published:** 2023-01-23

**Authors:** Yaning Shi, Neng Zhu, Yun Qiu, Junlan Tan, Feiying Wang, Li Qin, Aiguo Dai

**Affiliations:** 1 Laboratory of Stem Cell Regulation with Chinese Medicine and its Application, Hunan University of Chinese Medicine, Changsha, 410208 Hunan China; 2 Science and Technology Innovation Center, Hunan University of Chinese Medicine, Changsha, 410208 Hunan China; 3 Department of Urology, The First Hospital of Hunan University of Chinese Medicine, Changsha, 410021 Hunan China; 4 Hunan Provincial Key Laboratory of Vascular Biology and Translational Medicine, Changsha, 410208 Hunan China; 5 Department of Respiratory Diseases, Medical School, Hunan University of Chinese Medicine, Changsha, 410208 Hunan China; 6 Department of Respiratory Medicine, First Affiliated Hospital, Hunan University of Chinese Medicine, Changsha, 410021 Hunan China

**Keywords:** Resistin-like molecules, Inflammation, Proliferation, Parasite, Lung disease

## Abstract

Resistin-like molecules (RELMs) are highly cysteine-rich proteins, including RELMα, RELMβ, Resistin, and RELMγ. However, RELMs exhibit significant differences in structure, distribution, and function. The expression of RELMs is regulated by various signaling molecules, such as IL-4, IL-13, and their receptors. In addition, RELMs can mediate numerous signaling pathways, including HMGB1/RAGE, IL-4/IL-4Rα, PI3K/Akt/mTOR signaling pathways, and so on. RELMs proteins are involved in wide range of physiological and pathological processes, including inflammatory response, cell proliferation, glucose metabolism, barrier defense, etc., and participate in the progression of numerous diseases such as lung diseases, intestinal diseases, cardiovascular diseases, and cancers. Meanwhile, RELMs can serve as biomarkers, risk predictors, and therapeutic targets for these diseases. An in-depth understanding of the role of RELMs may provide novel targets or strategies for the treatment and prevention of related diseases.

**Video abstract**

**Supplementary Information:**

The online version contains supplementary material available at 10.1186/s12964-022-01032-w.

## Introduction

Resistin-like molecules (RELMs) are highly cysteine-rich proteins that include RELMα, RELMβ, Resistin and RELMγ [1, 2]. Holcomb et al. first discovered RELMα in bronchoalveolar lavage fluid (BALF) from mice with experimentally induced allergic pulmonary inflammation in 2000 and named it found in inflammatory zone 1 (FIZZ1). RELMs has a variety of nomenclature due to its discovery in different tissues and diseases (Table 1) [1, 3–8]. To date, four RELM proteins in rodents, including mRELMα, mRELMβ, mResistin and mRELMγ, and two RELM proteins in human, including hResistin and hRELMβ, have been identified [1–3, 8]. RELMα and RELMβ can regulate different physiological and pathological processes, including lung and intestinal inflammation, lung cell proliferation, glucose metabolism, skin and colon barrier defense, etc., and are related to the progression of multiple diseases such as lung diseases, intestinal diseases, cardiovascular diseases, and cancers. Resistin shares 59% identity at the amino acid level between human and mouse forms [9]. mResistin is almost exclusively expressed in white adipocytes of rodents, whereas macrophages are the primary source of hResistin in humans [10, 11]. Despite these differences between humans and rodents, accumulating evidence demonstrates the role of resistin as a mediator between inflammations and various chronic diseases such as metabolic disorders, cardiovascular diseases, and cancers [12, 13]. Here, we mainly elucidate the signaling pathways, biological functions, and related disease of the three isoforms of RELM family, including RELMα (Table 2), RELMβ (Table 3) and RELMγ.

**Table 1:** The nomenclature of RELMs

Rodent classification	Human classification
RELMα = FIZZ1 = HIMF = XCP2	Resistin = FIZZ3 = XCP1
RELMβ = FIZZ2 = XCP3	RELMβ = FIZZ2 = XCP2
Resistin = FIZZ3 = ADSF = XCP4	
RELMγ = FIZZ4 = XCP1	

*RELM* Resistin-like molecule, *FIZZ* Found in inflammatory zone, *HIMF* Hypoxia-induced mitogenic factor, *ADSF* Adipose tissue-specific secretory factor, *XCP* Ten-cysteine protein

**Table 2:** Diseases and related mechanisms regulated by RELMα

RELMs	Cell type	Animal type	Stimulator	Signaling pathways	Effector molecules	Effect	Diseases	References
RELMα	Rat PMVECs	C57BL/6 mice	NA	NA	VEGF↑, MCP-1↑, SDF-1↑ and VEGFR2↓	Proliferation and migration of PMVECs, and pulmonary inflammation	PH	[36]
RELMα	Macrophages of mice and HLFs	HIF-1α^+/+^ and HIF-1α^+/−^ mice	NA	HIF-1/VEGF-A/VEGFR2 pathway and IKK-β/NF-κB/HIF-1 pathway	HIF-1α↑, VEGF-A↑, VEGFR2↓, NF-κB↑ and IL-6↑	Pulmonary vascular remodeling, vascular tube formation and BMD cells recruitment and pulmonary inflammation	PH	[37]
RELMα	NA	RELMα^−/−^BALB/c mice	OVA	NA	(IL-1β, 1ra, 16, 17; CXCL1, 2, 9, 10, 13; MCP-1; M-CSF; TIMP-1 and TREM-1) ↑	Pulmonary vascular remodeling and pulmonary inflammation	PH	[38]
RELMα	Human macrophages	RELMα^−/−^C57BL/6 mice	NA	HMGB1/RAGE pathway	HMGB1↑ and RAGE↑	Pulmonary inflammation	PH	[39]
RELMα	Mouse PMVECs	IL-4 KO C57BL/6 mice	NA	IL-4/IL-4Ri pathway	VEGF↑, MCP-1↑ and SDF-1↑	Proliferation and migration of PMVECs	PH	[24]
RELMα	Human PMVECs and human PVSMCs	RELMα KO C57BL/6 J mice	NA	HMGB1/RAGE pathway	HMGB1↑, RAGE↑ and BMPR2↓	Autophagy, and anti-apoptosis and proliferation of PVSMCs	PH	[53]
RELMα	Human bone marrow MSCs	RELMα^−/−^ BALB/c mice	NA	PI3K/Akt pathway and ERK1/2 pathway	p-Akt↑ and p-ERK1/2↑	Proliferation of non-hematopoietic progenitor cells and MSCs	PH	[54, 55]
RELMα	Neonatal rat cardiomyocytes	SD rats	NA	PGC-1α/PPARα/ERRα pathway	PGC-1α↓, PPARα↓, ERRα↓, TFAM↓, Top1mt↓, POLG2↓, Polrmt↓, LCAD↓, VLCAD↓, ACADM↓, ACADS↓, Cpt-1a↓ and Cpt-1b↓	Mediating cardiac energy metabolism and mitochondrial structure, biogenesis and function	PH and right ventricular hypertrophy	[74]
RELMα	NA	*Retnla*-Tg mice	OVA	NA	Muc5ac↓, IL-4↓, IL-5↓, IL-13↓ and p-ERK1/2↓	Preventing allergic lung inflammation	Asthma	[78]
RELMα	Rat AECII and Rat lung fibroblasts	Wistar rats and Fisher 344 rats	OVA and BLM	NA	α-SMA↑and collagen type I↑	Myofibroblast differentiation	Pulmonary fibrosis and asthma	[76, 77]
RELMα	BECs	RELMα^−/−^ C57BL/6 mice	OSM	NA	COL1A1↑, COL3A1↑, MMP13↑ and TIMP1↑	ECM remodelling	Pulmonary fibrosis	[84]
RELMα	NA	SD Rat	L-arginine and sodium taurocholate	NA	p-Akt↑, p-NF-κB↑, p-p38 MAPK↑, p-ERK↑, ICAM-1↑, IL-1β↑, IL-6↑, IL-8↑, TNF-α↑ and CRP↑	Inflammation and lung injury	APALI	[40]
RELMα	NA	BALB/c mice and BL/6 mice	*N. brasiliensis*	NA	IL-17A↓	Limiting emphysema	Emphysema	[42]
RELMα	NA	RELMα^−/−^ C57BL/6 mice	*S. mansoni* eggs	BTK pathway	IL-4↓, IL-5↓ and IL-13↓	Limiting pulmonary inflammation	NA	[41]
RELMα	NA	RELMα^−/−^ and IL-17A^−/−^ C57BL/6 mice	C. rodentium	IL-23p19/IL-17A pathway	IL-23p19↑ and IL-17A↑	Intestinal inflammation	Intestinal inflammation	[45, 46]
RELMα	BMD macrophage	RELMα^−/−^ C57BL/6 or BALB/c mice	DSS	NA	IL-5↑, IL-6↑, IL-10↓, IL-17↑, TNF-α↑, CCL5↑, CCL11↑, p-NF-κB↑, p-ERK1/2↑ and p-p38 MAPK↑	Colitis	Colitis	[43, 44]
RELMα	NA	RELMα^−/−^ and RELMβ^−/−^ mice	*N. brasiliensis*	Fc receptor signaling and STAT6 pathway	IL-4↓, IL-13↓ and IL-17A↓	Downregulating lung inflammation and delaying parasite expulsion	Hookworm Infection	[87, 89–91]
RELMα	Murine myoblast cell and human endothelial progenitor cell	NA	NA	PDK1/PI3K/Akt/c-Jun pathway	p-PDK1↑, p-PI3K↑, p-Akt↑, p–c-Jun↑ and IL-18↑	Formation of endothelial progenitor cell tube and angiogenesis	Inflammatory myopathy	[106]
RELMα	Mouse eosinophils and mouse epithelial cells	CC10-rtTA-RELMα bitransgenic mice	DOX	NA	NA	Epithelial cell hyperplasia and basal layer thickness	EoE	[56]
RELMα	VSMCs of rat	ApoE^–/–^C57BL/6 J mice	High fat	NA	NA	Proliferation and migration of VSMCs	Atherosclerosis	[102]
RELMα	Murine AML-12 cells and peritoneal macrophage of mice	*Retnla*^−/−^ C57BL/6 J mice, *Ldlr*^−/−^*Retnla*^−/−^ mice and *Retnla*-Tg mice	High fat	Lrh-1/CYP7A1 pathway	Lrh-1↑, CYP7A1↑ and VLDL cholesterol↓	Increasing excretion of cholesterol in the form of bile acids	Hypercholesterolaemia and atherosclerosis	[20]
RELMα	Mouse macrophages	ApoE^−/−^ mice	Ang II	ERK1/2 pathway and JNK pathway	MCP-1↑, IL-6↑, MMP-2↑, MMP-9↑, p-ERK1/2↑ and p-JNK↑	Vascular inflammation	Abdominal aortic aneurysm	[107]
RELMα	Gastric cancer cells	Human	NA	NF-κB-MMP-9/VEGF pathway	NF-κB↑, VEGF↑ and MMP-9↑	Proliferation, migration and invasion of gastric cancer cells	Gastric cancer	[108]

**Table 3:** Diseases and related mechanisms regulated by RELMβ

RELMs	Cell type	Animal type	Stimulator	Signaling pathways	Effector molecules	Effect	Diseases	References
RELMβ	Human PASMCs	SD rats	NA	PI3K/Akt/mTOR pathway and PKC/MAPK pathway	Ca^2+^↑, p-PI3K↑, p-Akt↑, p-mTOR↑, p-PKC↑ and p-MAPK↑	PASMCs proliferation	PH	[28, 57]
RELMβ	Human PASMCs	NA	NA	KCNK3 pathway	KCNK3↓and p-STAT3↑	PASMCs proliferation	PH	[58]
RELMβ	Human PASMCs	NA	NA	FAK-survivin pathway	FAK↑ and survivin↑	PASMCs proliferation	PH	[59]
RELMβ	Human BECs	NA	NA	ERK1/2 pathway and PI3K/Akt pathway	p-ERK1/2↑, p-PI3K↑, p-Akt↑, TGF-β2↑, EGF↑, VEGF↑ and MUC5AC↑	BECs proliferation and airway remodelling	Asthma	[60]
RELMβ	Human lung fibroblasts	RELMβ^−/−^ C57BL/6 mice	NA	NA	TGF-β1↑, TGF-β2↑, collagen I↑, fibronectin↑, α-SMA↑, laminin α1↑, Hapl1↑ and p-ERK1/2↑	Proliferation of human lung fibroblasts and airway remodelling	Asthma	[61]
RELMβ	NIH/3T3 cell line and human lung fibroblasts	Mice deficient in STAT6 or IL-4 and IL-13 in the BALB/c background	OVA and *Aspergillus fumigatus*	IL-13 pathway and STAT6 pathway	IL-4↑, IL-13↑ and STAT6↑	Airway inflammation and lung remodeling	Asthma	[79]
RELMβ	NA	RELMβ^−/−^ C57BL/6 mice	*Aspergillus fumigatus*	NA	TNFα↓, VEGF↓, IFNγ↓ and IL-13↓	Preventing inflammation, goblet cell metaplasia, subepithelial fibrosis and airway resistance	Asthma	[81]
RELMβ	Human UVECs and human PAECs	NA	NA	TGF-β1/SMAD2/3/4 pathway	TGF-β1↑ and SMAD2/3/4↑	EndMT, proliferation and migration of human UVECs and human PAECs	Pulmonary fibrosis	[31]
RELMβ	Rat AECs, human airway epithelial cells and mouse lung fibroblast	RELMβ KO mice	BLM	STAT6 pathway and ERK pathway	Collagen type I↑ and α-SMA↑	Stimulation of fibroblast proliferation, promotion of myofibroblast differentiation, and the recruitment of BMD cells to the lung	Pulmonary fibrosis	[86]
RELMβ	Macrophage and CD4^+^ T cells	RELMβ^−/−^ mice	*Trichuris muris*	NA	TNF-α↑, IL-6↑, IL-12/23p40↑ and IFN-γ↑	Intestinal inflammation	Intestinal inflammation	[51]
RELMβ	NA	RELMβ^−/−^ mice	DSS	NA	NA	Colitis	Colitis	[47, 48]
RELMβ	NA	Muc2^−/−^/RELMβ^−/−^ C57BL/6 mice	NA	NA	RegIIIβ↑	Colitis and antibacterial	Colitis	[49]
RELMβ	NA	RELMβ^−/−^ C57BL/6 mice	*C. rodentium*	NA	IL-22↑	IECs proliferation and limiting the intestinal damage	Colitis	[63]
RELMβ	BMD macrophage of mice	SAMP1/YitFc mice	NA	NA	TNF-α↑, IL-6↑ and CCL5↑	Ileitis	Ileitis	[50]
RELMβ	Human colon cancer cells	C57BL/6 J mice	Trinitrobenzene sulfonic acid	PKC pathway and tyrosine kinases pathway	p-ERK1/2↑, MUC2↑ and M1/MUC5AC↑	Maintaining the mucosal defense barrier	Colitis	[70]
RELMβ	NA	IL-33–deficient C57BL/6 mice	*N. brasiliensis*	NA	IL-13↑ and IL-33↑	Recruiting eosinophils and eliminating parasite	Hookworm Infection	[94]
RELMβ	Human colon cancer cells	IL-4^–/–^ mice and IL-4Rα^–/–^ mice	Trichuris spiralis, N. brasiliensis, Trichinella muris and Strongyloides stercoralis	NA	IL-13↑	Inhibiting nematode chemotaxis	GI nematode infection	[95]
RELMβ	Human colon cancer cells	NA	*S. aureus*	NA	NA	Destroying the bacterial cytoplasm	S. aureus infection	[97]
RELMβ	Hepatocyteof mice	RELMβ transgenic C57BL/6 mice	High fat	MAPKs pathways	IRS-1↓, IRS-2↓, p-ERK1/2↑, p-p38 MAPK↑ and p-JNK↑	Hyperglycemia, hyperlipidemia, fatty liver, pancreatic islet enlargement and hepatic insulin resistance	Diabetes	[65]
RELMβ	NA	Wistar rats, C57BL/6 J mice	Glucose	PKC βII pathway and AMPK pathway	SGLT-1↓, GLUT2↑, PKC βII↑ and p-AMPK↑	Promotion of intestinal glucose absorption	Diabetes	[67]
RELMβ	Human aortic VSMCs	NA	High glucose	ERK1/2 pathway and p38 MAPK pathway	α-SMA↓, SM-MHC↓, calponin↓, OPN↑, p-ERK1/2↑ and p-p38 MAPK↑	VSMCs proliferation	Atherosclerosis	[62]
RELMβ	Macrophag from mice	RELMβ^−/−^ ApoE^−/−^ mice and RELMβ^−/−^ LDLR^−/−^ mice	High cholesterol	NA	VLDLR↑, SR-A1↑ ABCA1↑, ABCG1↓, TNFα↑, IL-1β↑, IL-6↑ and p-NF-κB↑	Formation of macrophage derived foam cell and inflammation	Atherosclerosis	[103]
RELMβ	Gastric carcinoma cells and normal gastric mucosa epithelial cells	NA	NA	NA	N-cadherin↑, Snail↑, Vimentin↑ and E-cadherin↓	Invasion and migration of gastric carcinoma cells	Gastric carcinoma	[110]
RELMβ	Macrophages of mice	RELMβ-KO mice	MCD and LPS	TLR4 pathway	TLR4↑, TNF-α↑, IL-1β↑ and IL-6↑	Lipid accumulation, inflammation and liver fibrosis	NASH	[120]

## Structure, distribution, and characteristics of RELMs

The mouse RELM gene and the human RELM gene are present on different chromosomes. The RELMβ gene (*Retnlb*), RELMα gene (*Retnla*), and RELMγ gene (*Retnlg*) in mice are in close proximity within one cluster and reside on chromosome 16 [2, 14]. Human *Retnlb* is located on chromosome 3q13.1 [5]. The RELM genes encode secreted proteins of 105–114 amino acids with three major domains: an amino (N) terminal signal sequence, a variable midsection, and a highly conserved carboxy (C) terminal signature sequence that constitutes nearly half of the molecule [1]. The C terminal of RELMs contains a unique and invariant spacing of the cysteine residues: C-X11-C-X8-CX-C-X3-C-X10-C-X-C-X-C-X9-CC-X3–6-END [1–3, 15]. In the whole members of the RELMs family, each of the 10 conserved cysteines participates in a conserved structure that constitutes the characteristic region. RELMβ is a disulfide-linked dimer, while RELMα is a monomer under non-reducing conditions. In RELMβ, the 11th cysteine mediates covalent dimerization through a disulfide bond, but this cysteine is absent in RELMα [16]. Furthermore, RELMβ can form hexamer, which consists of trimers linking to form hexamers through highly exposed disulfide bonds at the amino termini of their coiled-coil domains [17].

### RELMα

In homeostasis, *Retnla* is present in various tissues and organs, such as the lung, heart, tongue, breast tissue, and white adipose tissue [1, 18]. Among them, *Retnla* is most abundant in white adipose tissue, especially in gonadal fat, followed by subcutaneous fat, but at low levels in mammary tissue [18]. In addition, RELMα is hardly expressed in 3T3-L1 adipocytes and preadipocytes, suggesting that RELMα may be produced from the stromal vascular constituents of adipose tissue [1, 19, 20]. Numerous studies have reported that RELMα is expressed in macrophages, dendritic cells (DCs), type II alveolar epithelial cells (AEC II), and pulmonary microvascular endothelial cells (PMVECs), etc. [21–24].

The differential regulation of RELMα expression may depend on the relative expression levels of IL-4, IL-13, and their corresponding receptors such as IL-13Rα1. In response to IL-4, DCs can promote high-level production of RELMα in vitro and in vivo [21]. During T helper cell type 2 (Th2) priming, RELMα expression by DCs promotes the secretion of IL-10 and IL-13 by T cells [21]. In the lungs of mice, IL-13Rα1 significantly up-regulates RELMα expression following Aspergillus fumigatus allergen challenge [25]. Moreover, in a mouse model of acute pulmonary inflammation by ovalbumin (OVA) allergen challenge, the expression of genes encoding RELMα and RELMβ in the lung is induced with a signal transducer and activator of transcription 6 (STAT6)-dependent fashion [26]. The promoter region of RELMα contains functional binding sites for STAT6 and CCAAT/enhancer-binding protein (C/EBP) [26]. STAT6 directly regulates IL-4 and IL-13-triggered induction of RELMα expression at the transcriptional level by cooperating with C/EBP [26]. Meanwhile, IL-4 and IL-13 induce RELMα expression via activating STAT6 in rat AEC II during bleomycin (BLM)-induced lung fibrosis [22]. Previous studies have shown that paired immunoglobulin-like receptor B (PIR-B) negatively regulates IL-4-induced RELMα expression in the lungs of mice and suppresses IL-4-induced macrophage-derived RELMα in vitro [27]. Well-characterized markers of alternatively activated (M2) macrophages include RELMα and chitinase 3-like protein 3 (Ym1). In vivo induction of RELMα and Ym1 in macrophages from late-stage phospholipase C-deficient mutant *Trypanosoma brucei brucei-*infected mice depends on IL-4, whereas interferon-γ (IFN-γ) antagonizes the effect of IL-4 on the expression of RELMα and Ym1 in vitro [23].

### RELMβ

Generally, RELMβ is expressed in the lung, heart, kidney, and adrenal glands of human tissues, with the highest expression in the colon, while there is the little signal in the brain and liver [28]. Meanwhile, the mRNA and protein of RELMβ are abundant in the mouse colon and to a lesser level in the ileum [29]. A large number of studies have shown that RELMβ is expressed in goblet cells, pulmonary arteries smooth muscle cells (PASMCs), human umbilical vein endothelial cells (UVECs), human pulmonary artery endothelial cells (PAECs) and colon cancer cells, etc. [28, 30–32].

The expression of RELMβ is regulated by various signaling molecules. The region between -418 and -588 in the human RELMβ promoter contains two potential caudal type homeobox (Cdx) binding sites [32]. *Cdx2*, but not *Cdx1*, transactivates the human RELMβ promoter in a goblet cell-specific fashion in human colon cancer cells [32]. *Cdx2* participates in the induction of intestine-specific expression of RELMβ in the presence of commensal bacteria in mice [32]. Both RELMα and RELMγ are induced by Th2-mediated acquired immune responses, which are independent of Cdx2 [32]. The previous study has demonstrated that IL-4 and IL-13 protect against intestinal lumen-dwelling worms (expulsion of *Nippostrongylus brasiliensis* (*N. brasiliensis*) and *Heligmosomoides polygyrus* (*H. polygyrus*), but not *Trichinella spiralis*) primarily by inducing intestinal epithelial cells (IECs) to differentiate into goblet cells that secrete RELMβ [30]. In the intestines of mice, *Retnlb* expression is markedly inhibited by high-protein and high-carbohydrate diets [33]. Intervention with insulin and tumor necrosis factor-α (TNFα) as well as stearic acid (a saturated free fatty acid) upregulate RELMβ expression, while D-glucose downregulates RELMβ in human colon cancer cells [33]. However, galacto-oligosaccharides (GOS) enhance the expression of *Retnlb* in goblet cells [34]. In addition, deoxynivalenol suppresses RELMβ expression through activating protein kinase R (PKR) and mitogen-activated protein kinase (MAPK) p38, thereby inhibiting the mRNA expression of intestinal mucins (MUC1, MUC2, and MUC3) of goblet cells [35].

### RELMγ

To date, there are few studies on RELMγ. In mice, RELMγ mRNA and protein are typically abundant in bone marrow, lungs, colon, ileum, spleen, and pancreas [15, 29]. Especially in the bone marrow, about 30% of hematopoietic cells (including myelocytes and metamyelocytes or neutrophils) exist RELMγ. Furthermore, RELMγ is expressed in epithelial cells and goblet cells of the colon [29]. Increased serum concentrations of RELMγ are attributable to elevated production in the colon and bone marrow [29]. Interestingly, RELMβ and RELMγ form a homodimer and a heterodimer with each other in RELMs-overexpressing COS7 cells and mouse colon/serum [24]. Serum levels of RELMγ are obviously increased in high-fat-fed mice and db/db mice [29]. A previous study has shown that RELMγ enhances retinoic acid-induced proliferation rates and modulates terminal differentiation in the promyelocytic cell line HL60 [15].

## Biological functions mediated by RELMs

### RELMs are involved in the regulation of inflammation and immune responses

#### RELMα

**Fig. 1:**
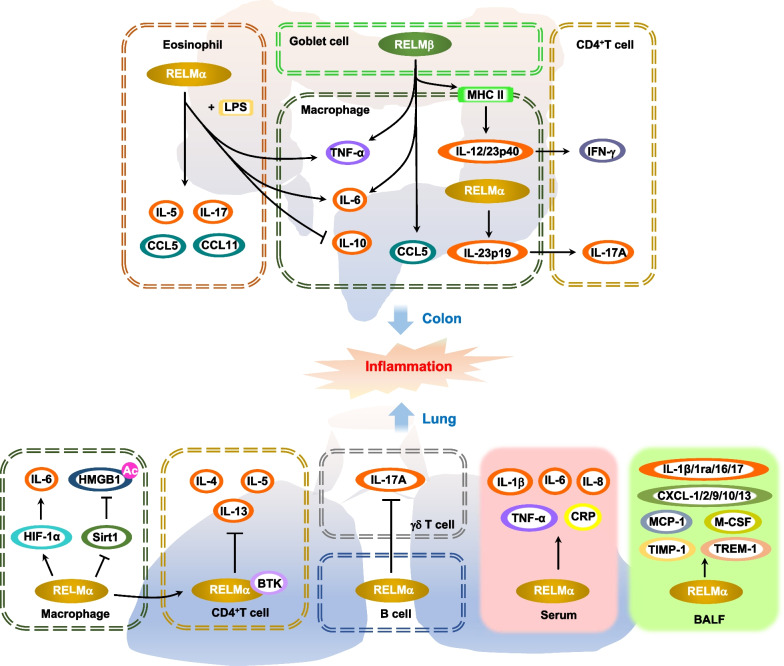
Signaling pathways of RELMα and RELMβ inducing the inflammation in the lung and colon. RELMα promotes IL-6 expression in macrophages in a HIF-1α-dependent manner. RELMα induces a series of inflammatory cytokines and chemokines such as interleukin IL-1β, -1ra, -16 and -17; CXCL-1, -2, -9, -10, -13; MCP-1; M-CSF; TIMP-1; and TREM-1 in BALF. RELMα induces acetylation of HMGB1 by inhibiting deacetylase Sirt 1, thereby enhancing vascular inflammation. RELMα promotes the release of inflammatory cytokines such as serum IL-1β, IL-6, IL-8, TNF-α, and CRP. M2 macrophage-derived RELMα binding to CD4^+^ T cells can attenuate lung inflammatory response by decreasing the production of Th2 cytokines (IL-4, IL-5, and IL-13) derived from CD4^+^ T cells in a BTK-dependent manner. Lung B cells can produce RELMα to downregulate IL-17A of γδ T cells, thereby limiting emphysema. RELMα exacerbates intestinal inflammation by promoting the IL-23p19/IL-17A immune axis. Eosinophils-derived RELMα promotes BMD macrophage activation by synergizing with LPS to amplify LPS-induced proinflammatory cytokine (IL-6 and TNF-α) secretion and suppresses anti-inflammatory cytokines (IL-10) production. RELMα induces proinflammatory eosinophil-directed cytokines (such as IL-5, CCL11, and CCL5) and IL-17. Goblet cell-derived RELMβ stimulates TNF-α, IL-6, and CCL5 in macrophages, thereby promoting intestinal inflammation. RELMβ-exposed macrophages induce expression of MHC II and secretion of IL-12/23p40, which can increase IFN-γ production by effector Th1 cells recruited to areas of inflammation

RELMα exhibits an intriguing regulatory role in lung inflammation (Fig. 1). For example, RELMα stimulates inflammation response by recruiting inflammatory cells such as macrophages in the lung, and these events are attenuated by vascular endothelial growth factor receptor-2 (VEGFR2) neutralization [36]. Meanwhile, RELMα promotes IL-6 expression in both macrophages and lung resident cells of the mouse lung in a hypoxia-inducible factor 1α (HIF-1α)-dependent manner [37]. In OVA-induced pulmonary vascular remodeling in mice, lack of RELMα obviously inhibits a series of inflammatory cytokines and chemokines such as interleukin (IL)-1β, -1ra, -16, and -17; chemokine (C-X-C motif) ligand (CXCL)-1, -2, -9, -10, -13; monocyte chemotactic protein-1 (MCP-1); macrophage colony-stimulating factor (M-CSF); tissue inhibitor of metalloproteinase 1 (TIMP-1); and triggering receptor expressed on myeloid cells 1 (TREM-1) in BALF [38]. In a mouse model of hypoxia, RELMα induces the expression of macrophage-specific high-mobility group box 1 (HMGB1), which belongs to damage-associated molecular pattern (DAMP) molecule, and receptor for advanced glycation end-products (RAGE) expression [39]. Notably, RELMα induces acetylation of HMGB1 by inhibiting the NAD^+^-dependent deacetylase sirtuin (Sirt) 1, which promotes nucleus-to-cytoplasm translocation and extracellular secretion of HMGB1, thereby enhancing vascular inflammation [39]. However, a deficiency of RELMα suppresses HMGB1/RAGE signals and reduces the number of macrophages, especially DAMP-producing macrophages in hypoxic lung tissue [39]. In severe acute pancreatitis (SAP) rats, over-expression of RELMα augments inflammatory activity by inducing the activation of protein kinase B (Akt), nuclear factor kappa-B (NF-κB), p38 MAPK, extracellular-signal-regulated kinases (ERK) and the expression of intracellular adhesion molecule 1 (ICAM-1) in lung tissue, and promoting the release of inflammatory cytokines such as serum IL-1β, IL-6, IL-8, TNF-α, and C-reactive protein (CRP), which aggravate acute pancreatitis-associated lung injury (APALI) [40]. In contrast, it has been reported that M2 macrophages-derived RELMα binding to CD4^+^ T cells can attenuate the magnitude of the *Schistosoma mansoni* (*S. mansoni*) eggs-induced lung inflammatory response by decreasing the production of Th2 cytokines (IL-4, IL-5, and IL-13) derived from CD4^+^ T cell in a BTK (as a binding partner for RELMα)-dependent manner [41]. Transient increases of IL-17A shortly after *N. brasiliensis* infection activates emphysema that impairs alveolar structures [42]. However, lung B cells can produce RELMα to downregulate IL-17A of γδ T cells, thereby limiting emphysema [42].

RELMα can modulate intestinal inflammation following infection (Fig. 1). During dextran sodium sulfate (DSS)-induced experimental colitis in mice, RELMα is highly expressed in eosinophils and colonic epithelial cells [43]. RELMα has also been found to promote bone marrow-derived (BMD) macrophage activation by synergizing with lipopolysaccharide (LPS) to amplify LPS-induced proinflammatory cytokine (IL-6 and TNF-α) secretion and suppresses anti-inflammatory cytokines (IL-10) production [43]. However, deficiency of RELMα protects against experimental colitis in mice [43]. RELMα establishes a novel link among colonic inflammation, energy uptake, and glucose metabolism. RELMα can be detected in serum, and its expression level is mediated by food intake and colonic inflammation [44]. Following DSS exposure, wild-type BALB/c and C57BL/6 mice display increased levels of circulating RELMα, whereas RELMα-deficient mice are distinctly protected from DSS-induced colitis and glucose injection-induced hyperglycemia independent of changes in insulin levels. Proinflammatory eosinophil-directed cytokines (such as IL-5, CC chemokine ligand 11 (CCL11)/eotaxin-1, and CCL5/RANTES) and IL-17 are substantially reduced in DSS-treated RELMα-deficient mice [44]. Consistently, DSS-treated RELMα-deficient mice displays significantly decreased eosinophil accumulation and reduced phosphorylation of NF-κB, ERK1/2, and p38 MAPK in macrophages and eosinophils [44]. After infection with *Citrobacter rodentium* (*C. rodentium*) in mice, a murine model for enteropathogenic *Escherichia coli* (EPEC)/enterohemorrhagic *Escherichia coli* (EHEC) intestinal diseases in humans, RELMα exacerbates intestinal inflammation through promoting the IL-23p19/IL-17A immune axis [45]. Intestinal epithelial cells, infiltrating macrophages, and eosinophils are potent sources of RELMα in the colon of *C. rodentium*-infected mice [45]. Genetic deletion of RELMα obviously alleviates infection-induced colitis in mice and shows the deficiency of IL-23p19 in macrophages as well as the decrease of IL-17A in CD4^+^ T cells [45]. Meanwhile, RELMα inhibits Th2 cells and M2 macrophages, which may induce Th17 immune response and intestinal inflammation [46].

#### RELMβ

RELMβ is predominantly expressed in goblet cells of the colon [47] and is involved in maintaining colonic barrier function and susceptibility to colonic inflammation (Fig. 1). Deletion of RELMβ dramatically alleviates goblet cell damage in DSS-induced colitis [48]. Muc2-deficient mice develop spontaneous colitis with marked induction of the goblet cell mediator RELMβ [49]. However, RELMβ deficiency dramatically ameliorates colitis development in *Muc2*^−/−^ mice [49]. Likewise, SAMP1/YitFc (SAMP1/Fc) mice develop spontaneous ileitis, which shares many characteristics with human Crohn’s disease. Early and rapid induction of ileal RELMβ expression is associated with the development and progression of inflammation in SAMP1/Fc mice [50]. And RELMβ is obviously expressed in most goblet cells, as well as some intermediate cells and Paneth cells located at the base of the ileal crypt epithelium in SAMP1/Fc mice [50]. Meanwhile, RELMβ stimulates naive BMD macrophages to secrete large amounts of levels of TNF-α, IL-6, and CCL5 [50]. In addition, RELMβ also promotes the expression of the inflammatory factors IL-8 and IL-1β by inducing phosphorylation of p38 MAPK in BECs, which is involved in airway inflammation in chronic obstructive pulmonary disease (COPD) [51]. After the mice are chronically infected with gastrointestinal (GI) helminth *Trichuris muris*, the goblet cell-derived RELMβ stimulates TNF-α and IL-6 in macrophages, thereby promoting intestinal inflammation [52]. The macrophages exposed to RELMβ induce the expression of major histocompatibility complex class II (MHC II) and the secretion of IL-12/23p40, which can increase IFN-γ production by recruiting effector Th1 cells into the inflammatory region [52]. The lack of RELMβ downregulates the expression of IFN-γ and TNF-α derived from parasite-specific CD4^+^ T cell, and attenuates intestinal inflammation in mice infected with *Trichuris muris* [52].

### RELMs participate in cell proliferation

#### RELMα

**Fig. 2:**
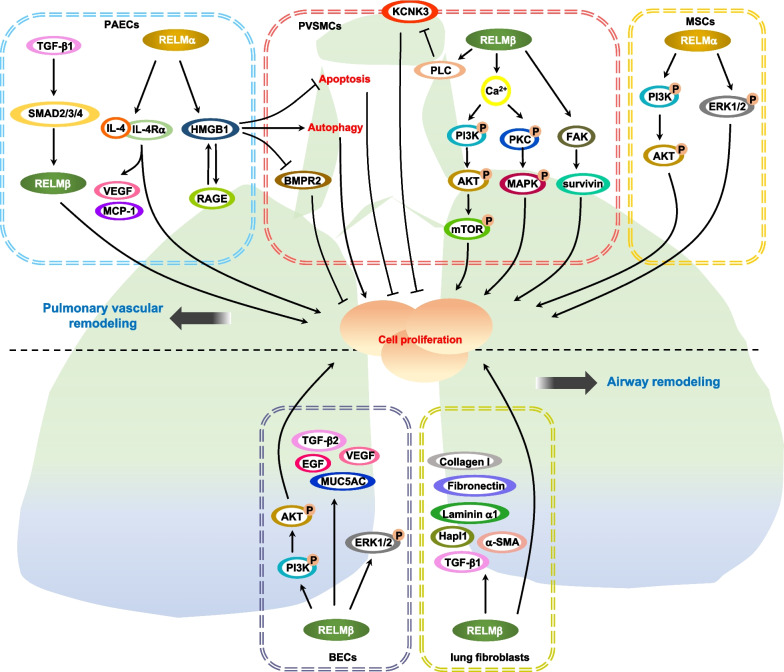
Signaling pathways of RELMα and RELMβ promoting PAH in the lung. RELMα facilitates vascular remodeling through IL-4/IL-4Rα signaling pathway to accelerate PMVECs proliferation, VEGF expression, and MCP-1 production. In PAH, HMGB1, which is produced and released by RELMα-stimulated ECs, leads to induction of autophagy and inhibition of apoptosis and BMPR2 expression in PVSMCs, thus reducing PVSMCs proliferation. ECs-derived HMGB1 also activates RAGE in ECs and PVSMCs to form a positive feedback loop, which contributes to the secretion and release of more HMGB1 and increases the expression of RAGE in these pulmonary vascular cells. RELMα induces robust proliferation of MSCs dependent on PI3K/Akt and ERK1/2 activation. RELMβ triggers PASMCs proliferation and pulmonary artery remodeling, resulting in PAH at least partially through Ca^2+^-dependent PI3K/Akt/mTOR pathway and protein kinase C (PKC)/MAPK pathway. RELMβ leads to PLC-mediated inhibition of KCNK3, thereby promoting PASMCs proliferation during PAH development. RELMβ promotes the proliferation of human PASMCs via the FAK-survivin pathway. In BECs, RELMβ increases cells proliferation through phosphorylation of ERK1/2, PI3K and Akt, and elevates the expression of a range of remodeling mediators, including TGF-β2, EGF, VEGF, and MUC5AC, which contribute to airway remodeling. RELMβ increases the production of TGF-β1, TGF-β2, collagen I, fibronectin, α-SMA, laminin α1, and Hapl1 as well as the proliferation of human lung fibroblasts, which have an important functional role in airway remodeling. TGF-β1 can trigger RELMβ transcription to promote EndMT, proliferation, and migration in human UVECs and human PAECs by activation of SMAD2/3/4

RELMα is involved in the proliferation of different cells (Fig. 2). In the pulmonary arteries of mice, RELMα induces proliferative activity, hypertrophy, collagen, and extracellular matrix (ECM) deposition in an IL-4-dependent manner [24]. And RELMα also increases the production of angiogenic factors/chemokines, including vascular endothelial growth factor (VEGF), MCP-1 and stromal derived factor-1 (SDF-1) in the lung resident cells, as well as macrophage infiltration, which are significantly inhibited in the lungs of IL-4-deficient mice [24]. Importantly, RELMα facilitates vascular remodeling through IL-4/IL-4Rα signaling pathway to accelerate PMVECs proliferation, VEGF expression and MCP-1 production [24]. In addition, VEGFR2 inhibitor suppresses RELMα-induced the proliferation and migration of PMVECs, as well as the production of MCP-1 and SDF-1 [36]. Pulmonary vascular remodeling has been reported to require RELMα/HMGB1/RAGE-driven endothelial cell (ECs)-pulmonary vascular smooth muscle cells (PVSMCs) crosstalk [53]. Particularly, in pulmonary arterial hypertension (PAH), as a key DAMP mediator, HMGB1, which is produced and released by RELMα-stimulated ECs, leads to induction of autophagy and inhibition of apoptosis and bone morphogenetic protein receptor 2 (BMPR2) expression in PVSMCs, thus reducing PVSMCs proliferation [53]. ECs-derived HMGB1 also activates RAGE in ECs and PVSMCs to form a positive feedback loop, which contributes to the secretion and release of more HMGB1 and increases the expression of RAGE in these pulmonary vascular cells [53]. A previous study has shown that pulmonary-specific overexpression of RELMα enhances the number of BMD cells recruited into the remodeling pulmonary vasculature [54]. Hypoxia, while stimulating RELMα expression, promotes the proliferation of non-hematopoietic progenitor cells in the lungs of mice, but not in lungs of RELMα knockout mice [55]. RELMα induces robust proliferation of mesenchymal stem cells (MSCs) dependent on phosphatidylinositol 3-kinase (PI3K)/Akt and ERK1/2 activation in vitro without affecting differentiation potential [55]. Moreover, in a mouse model of eosinophilic esophagitis (EoE), RELMα induction by doxycycline (DOX) in the esophagus can promote epithelial cell hyperplasia and basal layer thickness, recruit activated CD4^+^ and CD4^−^ T cell subsets, and exacerbate eosinophil accumulation [56].

#### RELMβ

RELMβ is closely associated with hypoxic-induced pulmonary vascular remodeling or hypoxia related fibrotic lung diseases (Fig. 2). Under hypoxic conditions, RELMβ mRNA is increased in lung epithelial cells, pulmonary artery adventitial fibroblasts and PASMCs [28]. RELMβ significantly promotes the proliferation of lung epithelial cells and PASMCs, which appear to be mediated through the PI3K pathway [28]. A previous study has reported that hypoxia-induced RELMβ triggers PASMCs proliferation and pulmonary artery remodeling, resulting in PAH at least partially through Ca^2+^-dependent PI3K/Akt/mTOR pathway and protein kinase C (PKC)/MAPK pathway [57]. Furthermore, hypoxia-induced RELMβ also leads to phospholipase C (PLC)-mediated inhibition of potassium channel subfamily K member 3 (KCNK3), thereby promoting PASMCs proliferation during PAH development [58]. RELMβ promotes the proliferation of human PASMCs via the focal adhesion kinase (FAK)-survivin pathway [59]. In human bronchial epithelial cells (BECs), IL-13-induced RELMβ increases cells proliferation through phosphorylation of ERK1/2, PI3K, and Akt, and elevates the expression of a range of remodeling mediators, including transforming growth factor (TGF)-β2, epidermal growth factor (EGF), VEGF and MUC5AC, which contribute to airway remodeling in diseases such as asthma [60]. In addition to epithelial cells, submucosal fibroblasts and endothelial structural cells, as well as macrophages and other infiltrating leucocytes are potential sources of RELMβ in human asthmatic airways [61]. RELMβ increases the production of TGF-β1, TGF-β2, collagen I, fibronectin, smooth muscle α-actin (α-SMA), laminin α1, and hyaluronan and proteoglycan link protein 1 (Hapl1) as well as the proliferation of human lung fibroblasts, which have an important functional role in airway remodeling [61]. Notably, TGF-β1 has been shown to trigger RELMβ transcription to promote endothelial-to-mesenchymal transition (EndMT), proliferation, and migration in human UVECs and human PAECs by activation of SMAD2/3/4 [31].

Vascular smooth muscle cells (VSMCs) proliferation is one of the key pathophysiological manifestations of atherosclerosis. RELMβ stimulates the migration and proliferation of VSMCs, and causes phenotypic modulation by downregulating the expressions of α-SMA, smooth muscle myosin heavy chain (SM-MHC) and calponin, and upregulating the expression of osteopontin (OPN) upon high glucose treatment, thereby inducing the occurrence and development of atherosclerosis [62]. Importantly, activation of ERK1/2 and p38 MAPK signaling pathways may be involved in VSMCs proliferation induced by RELMβ and high glucose co-stimulation [62]. In *C. rodentium*-induced colitis, goblet cell-derived RELMβ recruits CD4^+^ T cell to the infected intestine [63]. Upon reaching the intestine, CD4^+^ T cells produce the cytokine IL-22, which directly induces intestinal epithelial cells (IECs) proliferation to alleviate intestinal damage during *C. rodentium* infection [63].

#### RELMs regulate glucose metabolism

RELMα and RELMβ plays crucial roles in maintaining glucose metabolism and energy balance. CD301b protein is generally considered to be a prototypical marker of M2 macrophages [64]. Depletion of CD301b^+^ mononuclear phagocytes (MNPs) lead to reduced food intake, weight loss, lower blood glucose, increased insulin sensitivity, and a marked reduction in serum RELMα in mice [64]. However, reconstitution of RELMα restores body weight and normoglycemia in CD301b^+^ MNPs-depleted mice [64]. In addition, RELMβ has a similar effect on glucose metabolism. When fed a high-fat diet, transgenic mice with hepatic RELMβ over-expression exhibits obvious hyperglycemia, hyperlipidemia, fatty liver, pancreatic islet enlargement, and hepatic insulin resistance [65]. Meanwhile, the expression levels of insulin receptor substrate (IRS)-1 and IRS-2 proteins as well as insulin-induced PI3K and Akt are attenuated in RELMβ transgenic mice [65]. In hepatocytes, RELMβ significantly activates ERK1/2 and p38 MAPK, while weakly activates JNK [65]. Thus, chronic stimulation by RELMβ leads to glucose intolerance and hyperlipidemia associated with impaired insulin signaling, and the activations of the three MAPKs are probably related to suppression of insulin signaling [65]. At constant physiological insulin levels, elevated circulating RELMβ levels dramatically stimulates glucose production [66]. In the small intestine, transepithelial transport of glucose can be mediated by active absorption of sodium/glucose cotransporter 1 (SGLT-1) and by a diffusive component of aggregated glucose transporter 2 (GLUT2) at the apical membrane. RELMβ attenuates SGLT-1 activity, whereas enhancing the presence of GLUT2 in the brush border membranes (BBMs) of enterocytes [67]. It has been demonstrated that mucosal RELMβ can promote absorption of glucose in the jejunum of rat [67]. Luminal RELMβ can directly accelerate glucose transport by GLUT2 at BBMs by increasing protein kinase C βII and its translocation to the BBMs and phosphorylation of AMP-activated protein kinase (AMPK) [67].

#### The body barrier protection of RELMs

RELMα, expressed by epidermal keratinocytes and sebocytes, is currently believed to be an antimicrobial protein that shapes the composition of the skin microbiota and is required for vitamin-A-dependent resistance to skin infection [68]. RELMα is induced by microbiota colonization of murine skin, is bactericidal in vitro, and protects mouse skin from bacterial infection, which kills bacteria via membrane disruption [68].

RELMβ also participates in the local homeostatic regulation of the colonic epithelial barrier. Goblet cells are highly polarized exocrine epithelial cells that secrete proteins apically into the lumen of the small and large intestines, which contributes to the production and maintenance of protective mucus blankets by synthesizing and secreting high-molecular-weight glycoproteins named mucins [69]. Intestinal mucus secreted by goblet cells is mainly composed of MUC2 glycoprotein in humans and mice, and acts as a lubricant and a dynamic barrier to protect from the aggressive luminal environment [70]. In mice, *Retnlb* is uniquely restricted to the colonic crypt epithelium, and RELMβ protein is only expressed by goblet cells predominantly located in the distal half of the colon and cecum, with lower levels detectable in the proximal colon [71]. RELMβ enhances MUC2 and M1/MUC5AC gene expression in human colon cancer cells [70]. It also increases M1/MUC5AC secretion from human colon cancer cells and MUC2 secretion from murine intestinal goblet cells [70]. Intriguingly, RELMβ exerts its effect exclusively on the apical (luminal) side of human colon cancer cells, consistent with its role in luminal mucus secretion in mice [70]. Importantly, its action requires calcium, PKC, tyrosine kinases, and ERK activities, and acts synergistically with carbachol [70].

## RELMs are involved in multiple diseases

### RELMs and lung diseases

#### Pulmonary arterial hypertension

PAH is a vascular disorder with pulmonary vascular resistance and remodeling that can lead to right ventricular failure and death. In particular, PAH-induced pulmonary vascular remodeling is characterized by medial hypertrophy or hyperplasia, intimal and adventitial fibrosis, thrombogenesis, and plexiform lesions, as well as perivascular infiltration of inflammatory cells such as B- and T-lymphocytes, mast cells, dendritic cells, macrophages, etc. [72]. RELMα and RELMβ are involved in PAH-induced pulmonary vascular remodeling. OVA challenge-induced PAH promotes elevated secretion of RELMα, RELMβ, and RELMγ in BALF of wild-type mice [38]. RELMα induces pulmonary vascular remodeling, angiogenesis, and recruitment of BMD cells through a HIF-1α-dependent mechanism, thereby accelerating the development of PAH in mice [37]. In addition, the mechanism of RELMα-induced PAH is mediated, at least in part, by up-regulating lung VEGF-A expression and down-regulating VEGFR2 in a HIF-1α-dependent manner [37]. During OVA stimulation in mice, genetic ablation of *Retnla* attenuates vessel muscularization, suppresses perivascular inflammation, reduces the medial thickness of intra-alveolar vessels, and has fewer goblet cells in the upper airway epithelium, which prevents the increased pulmonary pressure and cardiac hypertrophy [38]. Knockdown of *Retnla* decreases genes associated with vascular remodeling (including those related to muscle proteins, contractile fibers, and the actin cytoskeleton) following the OVA challenge [38]. It has been found that intraperitoneal administration of N-acetylcysteine (NAC) prior to OVA challenge inhibits the expressions of RELMα, Ym1/chitinase 3-like protein 4 (Ym2), and surfactant-associated protein D (SP-D) in BALF and lung tissue of mice [73]. Furthermore, there is evidence that RELMα obviously regulates mitochondrial metabolic parameters (such as reducing basal and maximal respiration), signaling pathways (such as decreasing fatty acid oxidation (FAO) and increasing glycolytic oxidation), and bioenergetics (such as attenuating ATP-linked oxygen consumption rate (OCR), and inducing extracellular acidification rate (ECAR) and proton production) in the electron transport chain (ETC) of neonatal rat cardiomyocytes (NRCMs), thereby mediating cardiac energy metabolism and mitochondrial structure, biogenesis, and function, which is involved in pulmonary arterial hypertension and right ventricular hypertrophy [74]. Mechanistically, RELMα inhibits peroxisome proliferator-activated receptor gamma coactivator 1α (PGC-1α)/peroxisome proliferator-activated receptors alpha (PPARα)/estrogen-related receptor alpha (ERRα) signaling axis that decreases mitochondrial biogenesis genes (including mitochondrial transcription factor A (TFAM), mitochondrial topoisomerase I (Top1mt), mitochondrial DNA polymerase subunit gamma 2 (POLG2), and mitochondrial DNA-directed RNA polymerase (Polrmt)), FAO metabolic genes (including long-chain acyl-CoA dehydrogenase (LCAD), very long-chain acyl-CoA dehydrogenase (VLCAD), medium chain of acyl-CoA dehydrogenase (ACADM), and short chain of acyl-CoA dehydrogenase (ACADS)) as well as mitochondrial fatty acid (FA) transporter genes (including carnitine palmitoyltransferase-1A (Cpt-1a) and Cpt-1b) [74]. Moreover, RELMβ also regulates pulmonary hypertension. The expression of RELMβ is up-regulated in the lung tissue of patients with scleroderma-associated pulmonary hypertension [75]. RELMβ can promote the proliferation and activation of ERK1/2 in primary cultured human pulmonary endothelial and smooth muscle cells [75]. And, RELMβ induces the production of proinflammatory cytokine IL-6 by inducing the IκB kinase β (IKK-β)-NF-κB-HIF-1α axis in human primary lung fibroblasts (HLFs) [37].

#### Asthma and allergic lung diseases

Asthma is characterized by inflammation and structural changes in the lung. Most asthma is allergy-related, manifested as Th2-type inflammation, and also occurs as an immune response to parasites. During airway remodeling in a rat model of allergic pulmonary inflammation by OVA or BLM challenge, RELMα expression is induced in AEC II and stimulates fibroblasts differentiation into myofibroblasts that expresses differentiation markers such as α-SMA and collagen type I, which contribute to airflow obstruction during progressive airway remodeling [76, 77]. Conversely, RELMα overexpression has been reported to reduce the numbers of immune cells (including dendritic cells, macrophages, B cells, eosinophils, and neutrophils) in the BALF of OVA-challenged mice, and to reduce mucus production in the airway epithelium, concomitant with a down-regulated Muc5ac levels [78]. These processes are accompanied by decreased levels of Th2 cytokines, including IL-4, IL-5, and IL-13, whereas levels of OVA-specific immunoglobulin isotypes are unchanged [78]. Furthermore, RELMα overexpression attenuates allergic airway inflammation in OVA-challenged mice by inhibiting phosphorylation of ERK [78].

RELMβ has an important role in airway structural remodeling in asthma and allergic lung diseases. RELMβ is strongly produced in the lungs of mice with experimental asthma caused by multiple allergens (OVA and *Aspergillus*) and Th2 cytokines (IL-4 and IL-13) via IL-13- and STAT6-dependent mechanisms [79]. Following allergen challenge, RELMβ mRNA is induced in the airway epithelium and in infiltrative cells (mainly monocytes) surrounding blood vessels and airways [79]. In addition, RELMβ induces leukocyte accumulation (most prominently involving macrophages), goblet cell hyperplasia, perivascular and peribronchial collagen deposition, and fibroblast motogenic activity in lung [79]. Similarly, RELMβ is expressed in the human bronchial epithelium and that the immunoexpression is higher in asthmatics [80]. Contrarily, at homeostasis, loss of RELMβ up-regulates serum IgA and pro-inflammatory cytokines (TNFα, VEGF, and IFNγ) in the lung [81]. Inflammation and subepithelial fibrosis that characterize remodeling, as well as mediators such as IL-13, contribute to airway hyperresponsiveness (AHR) [81]. Nevertheless, the absence of RELMβ results in increased subepithelial fibrosis, AHR, and IL-13 expression in mice subjected to the fungal asthma model [81]. Cathelicidin antimicrobial peptide (CAMP), as a bactericidal agent in allergic asthma are also increased in the absence of RELMβ [81]. Deletion of RELMβ results in elevated markers of chronic diseases, including goblet cell numbers, Muc genes, airway wall remodeling, and hyperresponsiveness [81]. Thus, RELMβ may inhibit the development of chronic markers of allergic airways diseases. Studies have shown that MSC treatment can reduce airway inflammation, hyperresponsiveness and remodeling in chronic asthma [82]. Moreover, MSCs upregulate the RELMβ levels, which may serve as a biomarker of MSCs treatment outcomes [82].

#### Pulmonary fibrosis

Abnormal changes in the ECM in the airway or parenchymal tissue are pathological profiles of numerous respiratory diseases, including idiopathic pulmonary fibrosis (IPF), COPD, and asthma [83]. An oncostatin M (OSM)-RELMα pathway contributes to the ECM remodeling processes. Transient pulmonary over-expression of OSM by Adenovirus vector (AdOSM) markedly induces RELMα expression in mouse lung, increases RELMα in airway epithelial cells in vivo without IL-6 or STAT6, and can directly activate airway epithelial cells in vitro [84]. However, loss of RELMα leads to less accumulation of M2 macrophages, less increase of ECM remodeling genes (collagen Type I Alpha 1 (COL1A1), collagen type III alpha 1 (COL3A1), matrix metalloproteinase 13 (MMP-13), and TIMP-1), as well as less expression of parenchymal α-SMA in AdOSM-treated mice [84]. It has been shown that *Alternaria* facilitates STAT6-dependent acute airway eosinophilia and epithelial RELMα expression, thereby enhancing airway fibrosis and epithelial thickness [85]. Meanwhile, in BLM-treated mice, deficiency of PIR-B increases lung histopathology (such as excessive destruction of lung architecture, increased fibrocystic foci, and increased monocytes infiltration), and induces collagen expression and the IL-4-associated profibrogenic markers RELMα, MMP-12, TIMP-1 and osteopontin in alveolar macrophages, indicating that PIR-B can inhibit pulmonary fibrosis [27]. Furthermore, RELMβ is also involved in pulmonary fibrosis. RELMβ has been reported to be highly induced in the lungs of rodents with BLM-induced pulmonary fibrosis and human patients with idiopathic pulmonary fibrosis [86]. RELMβ expression is induced in both rat airway and alveolar epithelial cells as well as in human small airway epithelial cells, which is driven by Th2 cytokines (IL-4 and IL-13) through STAT6 signaling [86]. In vitro, RELMβ can stimulate the expression of collagen type I and α-SMA in lung fibroblasts, and promote fibroblast proliferation via activating ERK1/2 [86]. However, RELMβ deficiency significantly suppresses pulmonary fibrosis [86]. In addition, RELMβ has chemoattractant activity for lung recruitment of BMD cells, especially BMD CD11c^+^ dendritic cells [86].

### RELMs and infectious diseases

#### Parasitic infections

Acute infection with the GI nematode *N. brasiliensis* results in marked increases of RELMα and RELMβ levels systemically and in infected tissue [87]. Meanwhile, RELMα expression is highly elevated at the sites of parasite migration and residence during chronic infection with the filarial nematode *Litomosoides sigmodontis* [88]. RELMα but not RELMβ significantly affects the immune response to *N. brasiliensis* infection by down-regulating CD4^+^ Th2 adaptive immune response in the lung, thus protecting the host but improving parasite fitness [87]. While RELMα attenuates infection-induced inflammation, leading to an increased parasite burden, RELMβ has modest effects on acute lung inflammation and parasite burden [87]. Generally, in the lung, RELMα is mainly expressed in airway ECs and parenchymal cells. In the small intestine, RELMα is expressed by goblet cells in the basal crypts and circulating leukocytes in the submucosa [89]. In addition, alveolar macrophages are the primary source of immune cellular for RELMα in the lung, followed by dendritic cells and eosinophils [89]. During *N. brasiliensis* infection, RELMα in the airways is derived uniquely by non-immune cells, whereas immune cells are the major source of systemic RELMα in serum [89]. Although RELMα is highly expressed by non-BM-derived airway ECs and BM-originated immune cells, immune cells-derived RELMα is essential and sufficient for reducing the *N. brasiliensis* immune responses, while non-BM-derived RELMα has no obvious effect on *N. brasiliensis* infection [89]. Macrophages expressing RELMα are vital for suppressing lethal lung injury during primary *N. brasiliensis* infection [90]. RELMα acts as an immune brake that provides mutually beneficial effects on the host and parasite by preventing tissue damage and delaying parasite expulsion [89]. RELMα produced by BM-derived macrophages attenuates the Th2 inflammatory immune response induced by *N. brasiliensis* and subsequent *N. brasiliensis* clearance partly by direct inhibition of macrophage recruitment and macrophage-worm interactions [89]. It has been shown that RELMα^−/−^ mice infected with the GI parasite *N. brasiliensis* exacerbate lung pathology to migrating larvae, reduced fecundity, and facilitated expulsion of adult worms from the intestine, suggesting enhanced Th2 immunity [91]. Furthermore, there is evidence that RELMα-expressing lung interstitial but not alveolar macrophages are increased in a STAT6-dependent manner during primary *N. brasiliensis* infection [90]. During *N. brasiliensis* secondary challenge, RELMα-expressing macrophages provide protective immunity against migrating parasites [90]. The formation of primary and secondary pulmonary granuloma is exacerbated in RELMα-deficient mice with the eggs of helminth parasite *S. mansoni* challenge, and the number of granuloma-associated eosinophils and serum IgE titers are also elevated [91]. Moreover, RELMα-deficient mice significantly increase hepatic granulomatous inflammation as well as the development of fibrosis and progression to hepatosplenic disease in mice chronically infected with *S. mansoni* cercariae [91]. The expression of RELMα is dependent on IL-4 and IL-13 and is inhibited by IFN-γ, and eosinophils and epithelial cells are the major producers of RELMα in the liver and lung, respectively [91]. The Th2-inducible gene RELMα suppresses resistance to GI nematode infection, pulmonary granulomatous inflammation, and fibrosis by negatively regulating Th2-dependent responses [91].

Increased numbers of goblet cells are characteristic of infection with the GI nematode parasite *N. brasiliensis* and *H. polygyrus*, and are a source of protective factors, such as RELMβ, that are critical for worm expulsion [30]. The expression of *Retnlb* in bronchial epithelium is up-regulated after *N. brasiliensis* infection in parallel with goblet cell hyperplasia [92]. However, goblet cell numbers and RELMβ expression are decreased significantly in IL-13Rα1^−/−^ mice following secondary infection with the GI nematode parasite *Heligmosomoides bakeri* [93]. The binding of IL-13 to IL-13Rα1 is crucial for goblet cell proliferation, and enhanced RELMβ expression may be correlated with an increased number of goblet cells [93]. Within hours of primary *N. brasiliensis* infection, the release of IL-33 drives the initial expansion of IL-13^+^ innate lymphoid type 2 cells (ILC2s)/nuocytes, followed by IL-13^+^ CD4^+^ T cells in three days [94]. This accumulation of IL-13 production contributes to IECs generating RELMβ and recruits eosinophils, which together lead to parasite destruction [95]. Furthermore, activation of RELMβ is a highly specific Th2 cytokine (IL-13)-dependent intestinal response that is mediated by exposure to phylogenetically and biologically distinct GI nematode parasites (*Trichuris spiralis*, *N. brasiliensis*, *Trichinella muris* and *Strongyloides stercoralis*) that reside in different regions of the GI tract [95]. RELMβ is a goblet cell-specific immune effector molecule in the expulsion of GI nematodes by disrupting the ability of nematodes to optimally sense the GI microenvironment [95].

#### Bacterial infections

Elevated levels of RELMβ are closely related to the severity and prognosis of disease in patients with community-acquired pneumonia (CAP) [96]. Serum RELMβ levels are significantly increased in patients with severe CAP, particularly in non-survivors [96]. Meanwhile, the serum RELMβ level in patients with bacterial infection is notably higher than that in patients with non-bacterial infection [96]. However, the RELMβ level in the *Mycoplasma pneumonia*-positive group is significantly lower than that in the *Mycoplasma pneumonia*-negative group [96]. Elevated levels of RELMβ displays positive correlations with the pneumonia severity index (PSI) and CURB-65 [96]. RELMβ in serum of patients with CAP is associated with 30-day mortality outcome; and the combination of clinical severity score and RELMβ significantly improve mortality predictive ability [96].

RELMβ is also a colonic antimicrobial protein. The amount of RELMβ in healthy human feces is comparable to that showing antimicrobial activity in vitro, indicating that RELMβ may be involved in the regulation of gut microflora [97]. The mRNA and protein expression of RELMβ are induced by heat-inactivated *S. aureus*, but not by *Escherichia coli* in LS174T colonic epithelial cells [97]. RELMβ thus reveals antimicrobial activity against *Staphylococcus aureus* (*S. aureus*), including methicillin-resistant *S. aureus* (MRSA) [97]. Mechanistically, RELMβ binds to the cell surface of *S. aureus* and subsequently destroys the bacterial cytoplasm [97]. It has been reported that mouse and human RELMβ selectively kills Gram-negative bacteria by forming a membrane-permeabilized pores that lyses the targeted bacterial cells [98]. In mice, RELMβ restricts the entry of *Proteobacteria* into the inner mucus layer of the colon, thereby limiting bacterial contact with the colonic mucosal surface [98]. The mucus produced by goblet cells contributes to the barrier function of the gut. Generally, mice lacking Muc2 develop spontaneous colitis [49]. RELMβ expression in *Muc2-*deficient mice significantly stimulates secretion of the antimicrobial lectin RegIIIβ that exerts its microbicidal effect predominantly on Gram-positive *Lactobacillus* species, which leads to microbial dysbiosis that exacerbates colitis [49]. Furthermore, oral supplementation with murine *Lactobacillus* spp. attenuates spontaneous colitis in concert with increased production of short-chain fatty acids in *Muc2*^−/−^ mice [49]. In the absence of colonic fibroblasts, the lactic acid bacteria (LAB) (including *Lactobacillus acidophilus* CCFM137, *Streptococcus thermophilus* CCFM218, *Lactobacillus reuteri* CCFM14, and *Lactobacillus rhamnosus* CCFM237) increases mucus-related genes *Retnlb* transcription in goblet cells [99]. Nevertheless, none of the aforementioned LAB strains increases *Retnlb* expression in the presence of fibroblasts [99]. More importantly, TNF-α and IL-13 inhibit *Retnlb* expression under LAB strains [99, 100]. Furthermore, RELMβ increases the production of IL-2 and IL-6 by three pathogens (EPEC, *C. rodentium*, and *Cryptosporidium parvum* (*C. parvum*)) and induces both cytokines in the absence of pathogens [101].

### RELMs and cardiovascular diseases

RELMα and RELMβ plays an importance role in the pathology of atherosclerosis. RELMα is up-regulated in atherosclerotic plaque of ApoE^−/−^ mice [102]. Importantly, RELMα dramatically enhances the proliferation and migration of VSMCs [102]. It has been demonstrated that RELMα ameliorates HFD-induced hypercholesterolaemia and atherosclerosis by promoting the conversion of cholesterol into bile acids and mediating its subsequent fecal excretion via liver receptor homologue-1 (Lrh-1)-induced enhancement of hepatic cholesterol 7α-hydroxylase (CYP7A1) gene transcription [20]. Furthermore, RELMβ accelerates atherosclerosis development through lipid accumulation and inflammatory facilitation. Serum levels of RELMβ and RELMγ are obviously increased in high-fat-fed mice and db/db mice [29]. Enhanced serum concentrations of RELMβ and RELMγ are attributable to elevated production in the colon (both RELMβ and RELMγ) and bone marrow (RELMγ only) [29]. RELMβ is abundantly expressed in foam cells of the human coronary artery atherosclerotic lesions [103]. RELMβ induces the formation of macrophage-derived foam cells by triggering lipid accumulation and increases the expressions of very low-density lipoprotein receptor (VLDLR), scavenger receptor A1 (SR-A1) and ATP binding cassette transporter A1 (ABCA1), as well as decreases the expressions of ABCG1 [103]. Furthermore, RELMβ up-regulates the expressions of inflammatory cytokines (such as TNFα, IL-1β, and IL-6) and NF-κB pathways with LPS stimulation in macrophages [103].

RELMα and RELMβ are also involved in other vascular diseases. Idiopathic inflammatory myopathies are a rare and heterogeneous group of acquired autoimmune muscle disorders [104]. High levels of serum IL-18 have been observed in patients with inflammatory myopathy [105]. In addition to its pro-inflammatory effects, IL-18 is a potent angiogenic mediator. There is evidence that RELMα promotes IL-18 secretion in myoblasts and induces endothelial progenitor cell tube formation and angiogenesis through activating 3-phosphoinositide-dependent protein kinase-1 (PDK1)/PI3K/Akt/c-Jun signaling pathway [106]. Moreover, deletion of RELMβ inhibits angiotensin II (Ang II)-induced abdominal aortic aneurysm (AAA) formation in ApoE^−/−^ mice [107]. The underlying mechanism may involve the down-regulation of pro-inflammatory cytokines (MCP-1 and IL-6), MMP-2 and MMP-9, which are mediated by phosphorylation of ERK1/2 and JNK [107].

### RELMs and cancers

The RELM family is strongly associated with the occurrence and progression of cancers. The expressions of RELMα and RELMβ are related to the clinicopathological parameters and prognosis of gastric cancer. The up-regulation of RELMα in gastric cancer tissues is positively correlated with tumor size, clinical stage and promotes gastric cancer through angiogenesis [108]. Silencing of RELMα expression significantly inhibits proliferation, migration and invasion in gastric cancer cells, and prevents NF-κB activation and attenuates VEGF and MMP-9 expressions [109]. In addition, RELMβ has been found to be absent in the normal gastric mucosa and aberrantly expressed in a majority of human gastric cancer tissues, with expression restricted to the cytoplasm of cancer cells and goblet cells of intestinal metaplasia [109]. RELMβ is positively correlated with tumor differentiation in gastric cancer and negatively associated with lymph node metastasis, tumor infiltration, and heparanase expression, independent of age, gender, tumor location and size, tumor-node metastasis stages, and Ki-67 expression [109]. Furthermore, patients with positive RELMβ expression has markedly longer overall survival than those with negative expression [109]. Studies have shown that RELMβ is abundantly expressed in gastric carcinoma cells, and over-expression of RELMβ can promote the invasion and migration of gastric carcinoma cells via facilitating EMT, as evidenced by EMT-related proteins, such as up-regulation of N-cadherin, Snail, Vimentin and down-regulation of E-cadherin [110]. Persistent infection with the Gram-negative bacterial pathogen *Helicobacter pylori* (*H. pylori*) induces chronic gastric inflammation, which is the most critical risk factor for the development of adenocarcinoma. *H. pylori*-induced RELMβ is also involved in the pathogenesis of gastric cancer [111, 112]. It has been found that higher expression of RELMβ is observed in *H. pylori*-positive intestinal metaplasia, dysplasia, intestinal-type and diffuse-type gastric cancers, whereas elimination of *H. pylori* significantly attenuate RELMβ expression in intestinal metaplasia [113]. The development of goblet cells, a feature of intestinal metaplasia in Barrett’s esophagus (BE), is a sentinel event leading to an increased risk of adenocarcinoma, which is an incidence 30 to 125 times that of the general population [105, 114]. RELMβ expression is restricted to goblet cells in the metaplastic epithelium of the distal esophagus in patients with BE, not in gastric-type mucosa or squamous epithelium, and is enhanced in dysplasia, which can be used as a potential biomarker for the accurate diagnosis of BE. Moreover, the expression of CDX-2 is mainly localized to the goblet cells of intestine metaplasia, and is positively correlated with RELMβ expression, indicating that CDX-2 may regulate the expression of RELMβ in BE [116].

The intestinal epithelium is a key interface between the gut luminal contents and the human internal environment, which responds to the luminal environment and internal stimuli by producing proteins that are secreted at both the apical and basolateral sides [117]. RELMβ is positively associated with smoking and negatively associated with physical activity, both of which are risk factors for colon cancer, suggesting that RELMβ may be participated in regulating the effects of these two lifestyle factors on risk of colon cancers [118]. It has been shown that RELMβ is over-expressed in most human colon cancer tissues, and the expression is restricted to goblet cells in the colonic epithelium [117]. However, the mean postoperative survival time of RELMβ-positive patients is obviously longer than that of RELMβ-negative patients [117]. Moreover, RELMβ expression is remarkably correlated with the expression of the transcription factor caudal-type homeobox protein 2 (CDX-2), but not with that of proliferative index Ki-67 [117]. RELMβ positivity in colon cancer is associated with histological grade of differentiation and lymph node metastasis, but not with age, gender, tumor location and size, tumor infiltration, Dukes’ stage, venous invasion, and liver metastasis [117]. These indicate that RELMβ expression is correlated with clinicopathological parameters and prognosis of colon cancers [117].

### RELMs and other diseases

RELMα may play a regulatory role in insulin-resistance-mediated gallbladder dyskinesia. RELMα enhances insulin resistance and reduces optimal gallbladder tension in response to acetylcholine in C57BL/6 J lean non-diabetic mice, but does not affect gallbladder response to neuropeptide Y or cholecystokinin [119]. Furthermore, RELMβ is a potential novel target for non-alcoholic steatohepatitis (NASH) therapy. The expression of RELMβ is obviously induced in colon and liver kupffer cells by methionine-choline deficient (MCD) diet feeding [120]. RELMβ deficiency attenuates the development of MCD diet-induced NASH by suppressing lipid accumulation, inflammation, and liver fibrosis [120]. Furthermore, RELMβ deficiency decreases serum LPS concentrations and inflammatory cytokine productions (TNF-α, IL-1β, and IL-6) in response to LPS by downregulating toll-like receptor 4 (TLR4) signaling in the liver [120]. *Lactobacillus* (*L. gasseri* and *L. reuteri*) are increased in RELMβ-deficient mice following MCD diet feeding, which may be involved in the protection from impaired gut permeability [120]. Moreover, RELMβ is secreted by the inner enamel epithelium and localized at the lower edge of the dentin surface facing the Hertwig’s epithelial root sheath (HERS) and dental follicle in rats, which might be involved in cementogenesis [121].

**Fig. 3:**
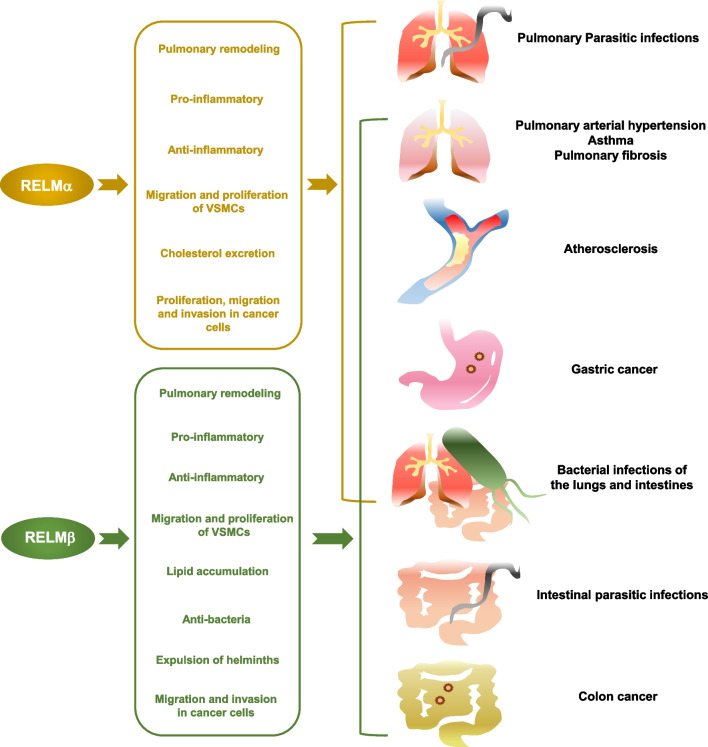
RELMα and RELMβ participate in various diseases

Therefore, RELMα and RELMβ have variable effects during the development of multiple diseases (Fig. 3).

## Conclusions

In the RELM family, RELMα, RELMβ, and RELMγ are distributed in different tissues and cells, which have distinct biological functions including inflammatory response, cell proliferation, glucose metabolism, and body barriers, etc., and are involved in the regulation of different diseases such as lung diseases, intestinal diseases, cardiovascular diseases, and tumors and so on. To date, there are numerous studies on RELMα and RELMβ, while RELMγ is relatively less. RELMs can mediate various signaling pathways, forming a complex network that regulates multiple physiology and pathology. However, due to the different effects of RELMs in mediating the occurrence and development of multiple diseases, the advantages and disadvantages of RELMs cannot be simply defined. RELMα participates in pulmonary hypertension but attenuates intestinal parasite infection. Additionally, RELMβ promotes gastric cancer development but inhibits bacterial infection in the gut. An in-depth understanding of the role of RELMs is essential for sorting out their related signaling pathways, revealing the molecular regulation of related diseases, and finding clinical treatments for the diseases. Therefore, the RELM family has potential value in clinical application, which can treat the associated diseases, and can be used as a marker to indicate the type and degree of diseases. However, the current studies on the RELMs field are still weak, and the functional effects remain controversial. The exploration of RELMs receptors and direct target proteins is required to be strengthened, which contributes to elucidating more biological functions of RELMs and discovering valuable insights into the pathogenesis of related diseases. How to develop drugs targeting RELMs will be helpful for the clinical treatment of diseases. More hidden physiological and pathological effects and underlying mechanisms of RELMs need to be further investigated. It is worth noting that RELMα and RELMγ are absent in the human body, whether the reason can be explored using the theory of species evolution to explain the relationship between RELMs and humans or other species. The current studies mainly focus on RELMα and RELMβ, while research on RELMγ needs to be further developed. Therefore, this review provides a thorough understanding of the physiological and pathological functions and mechanisms of RELMs, opens a door for the prevention and treatment of inflammation-related diseases, cardiovascular diseases, cancers, etc., and also contributes to the direction for the future development of RELMs.

## Data Availability

Not applicable.

## Abbreviations

RELMs: Resistin-like molecules
BALF: Bronchoalveolar lavage fluid
FIZZ1: Found in inflammatory zone 1
DCs: Dendritic cells
AEC II: Type II alveolar epithelial cells
PMVECs: Pulmonary microvascular endothelial cells
Th2: T helper cell type 2
OVA: Ovalbumin
STAT6: Signal transducer and activator of transcription 6
C/EBP: CCAAT/enhancer-binding protein
BLM: Bleomycin
PIR-B: Paired immunoglobulin-like receptor B
M2: Alternatively activated
Ym1: Chitinase 3-like protein 3
IFN-γ: Interferon-γ
PASMCs: Pulmonary arteries smooth muscle cells
UVECs: Umbilical vein endothelial cells
PAECs: Pulmonary artery endothelial cells
Cdx: Caudal type homeobox
N. brasiliensis: Nippostrongylus brasiliensis
H. polygyrus: Heligmosomoides polygyrus
IECs: Intestinal epithelial cells
TNF-α: Tumor necrosis factor-α
GOS: Galacto-oligosaccharides
PKR: Protein kinase R
MAPK: Mitogen-activated protein kinase
MUC: Mucin
VEGFR2: Vascular endothelial growth factor receptor-2
HIF-1α: Hypoxia-inducible factor 1α
IL: Interleukin
CXCL: Chemokine (C-X-C motif) ligand
MCP-1: Monocyte chemotactic protein-1
M-CSF: Macrophage colony-stimulating factor
TIMP-1: Tissue inhibitor of metalloproteinase 1
TREM-1: Triggering receptor expressed on myeloid cells 1
HMGB1: High-mobility group box 1
DAMP: Damage-associated molecular pattern
RAGE: Receptor for advanced glycation end-products
Sirt: Sirtuin
SAP: Severe acute pancreatitis
Akt: Protein kinase B
NF-κB: Nuclear factor kappa-B
ERK: Extracellular-signal-regulated kinases
ICAM-1: Intracellular adhesion molecule 1
CRP: C-reactive protein
APALI: Acute pancreatitis-associated lung injury
S. mansoni: Schistosoma mansoni
C. rodentium: Citrobacter rodentium
EPEC: Enteropathogenic *Escherichia* coli
EHEC: Enterohemorrhagic *Escherichia* coli
DSS: Dextran sodium sulfate
BMD: Bone marrow-derived
LPS: Lipopolysaccharide
CCL11: CC chemokine ligand 11
GI: Gastrointestinal
MHC II: Major histocompatibility complex class II
ECM: Extracellular matrix
VEGF: Vascular endothelial growth factor
SDF-1: Stromal derived factor-1
ECs: Endothelial cell
PVSMCs: Pulmonary vascular smooth muscle cells
PAH: Pulmonary arterial hypertension
BMPR2: Bone morphogenetic protein receptor 2
MSCs: Mesenchymal 36 stem cells
PI3K: Phosphatidylinositol 3-kinase
EoE: Eosinophilic esophagitis
DOX: Doxycycline
PKC: Protein kinase C
PLC: Phospholipase C
KCNK3: Potassium channel subfamily K member 3
FAK: Focal adhesion kinase
BECs: Bronchial epithelial cells
TGF: Transforming growth factor
EGF: Epidermal growth factor
α-SMA: Smooth muscle α-actin
Hapl1: Hyaluronan and proteoglycan link protein 1
EndMT: Endothelial-to-mesenchymal transition
VSMCs: Vascular smooth muscle cells
SM-MHC: Smooth muscle myosin heavy chain
OPN: Osteopontin
IECs: Intestinal epithelial cells
MNPs: Mononuclear phagocytes
IRS: Insulin receptor substrate
SGLT-1: Sodium/glucose cotransporter 1
GLUT2: Glucose transporter 2
BBMs: Brush border membranes
AMPK: AMP-activated protein kinase
Ym2: Chitinase 3-like protein 4
SP-D: Surfactant-associated protein D
FAO: Fatty acid oxidation
OCR: Oxygen consumption rate
ECAR: Extracellular acidification rate
ETC: Electron transport chain
NRCMs: Neonatal rat cardiomyocytes
PGC-1α: Peroxisome proliferator-activated receptor gamma coactivator 1α
PPARα: Peroxisome proliferator-activated receptors alpha
ERRα: Estrogen-related receptor alpha
TFAM: Mitochondrial transcription factor A
Top1mt: Mitochondrial topoisomerase I
POLG2: Mitochondrial DNA polymerase subunit gamma 2
Polrmt: Mitochondrial DNA-directed RNA polymerase
LCAD: Long-chain acyl-CoA dehydrogenase
VLCAD: Very long-chain acyl-CoA dehydrogenase
ACADM: Medium chain of acyl-CoA dehydrogenase
ACADS: Short chain of acyl-CoA dehydrogenase
FA: Fatty acid
Cpt-1a: Carnitine palmitoyltransferase-1A
IKK-β: IκB kinase β
HLFs: Human primary lung fibroblasts
AHR: Airway hyperresponsiveness
CAMP: Cathelicidin antimicrobial peptide
IPF: Idiopathic pulmonary fibrosis
COPD: Chronic obstructive pulmonary disease
OSM: Oncostatin M
COL1A1: Collagen type I Alpha 1
COL3A1: Collagen type III alpha 1
MMP-13: Matrix metalloproteinase 13
ILC2s: Innate lymphoid type 2 cells
CAP: Community-acquired pneumonia
PSI: Pneumonia severity index
S. aureus: Staphylococcus aureus
LAB: Lactic acid bacteria
C. parvum: Cryptosporidium parvum
Lrh-1: Liver receptor homologue-1
CYP7A1: Cholesterol 7α-hydroxylase
VLDLR: Very low-density lipoprotein receptor
SR-A1: Scavenger receptor A1
ABCA1: ATP binding cassette transporter A1
PDK1: 3-Phosphoinositide-dependent protein kinase-1
Ang II: Angiotensin II
AAA: Abdominal aortic aneurysm
H. pylori: Helicobacter pylori
BE: Barrett’s esophagus
CDX-2: Caudal-type homeobox protein 2
NASH: Non-alcoholic steatohepatitis
MCD: Methionine-choline deficient
HERS: Hertwig’s epithelial root sheath

## References

[1] Steppan CM, Brown EJ, Wright CM, Bhat S, Banerjee RR, Dai CY, Enders GH, Silberg DG, Wen X, Wu GD (2001). A family of tissue-specific resistin-like molecules. Proc Natl Acad Sci U S A.

[2] Gerstmayer B, Küsters D, Gebel S, Müller T, Van Miert E, Hofmann K, Bosio A (2003). Identification of RELMgamma, a novel resistin-like molecule with a distinct expression pattern. Genomics.

[3] Holcomb IN, Kabakoff RC, Chan B, Baker TW, Gurney A, Henzel W, Nelson C, Lowman HB, Wright BD, Skelton NJ (2000). FIZZ1, a novel cysteine-rich secreted protein associated with pulmonary inflammation, defines a new gene family. Embo j.

[4] Steppan CM, Bailey ST, Bhat S, Brown EJ, Banerjee RR, Wright CM, Patel HR, Ahima RS, Lazar MA (2001). The hormone resistin links obesity to diabetes. Nature.

[5] Fan C, Johns BA, Su Q, Kolosova IA, Johns RA (2013). Choosing the right antibody for resistin-like molecule (RELM/FIZZ) family members. Histochem Cell Biol.

[6] Chumakov AM, Kubota T, Walter S, Koeffler HP (2004). Identification of murine and human XCP1 genes as C/EBP-epsilon-dependent members of FIZZ/Resistin gene family. Oncogene.

[7] Kim KH, Lee K, Moon YS, Sul HS (2001). A cysteine-rich adipose tissue-specific secretory factor inhibits adipocyte differentiation. J Biol Chem.

[8] Teng X, Li D, Champion HC, Johns RA (2003). FIZZ1/RELMalpha, a novel hypoxia-induced mitogenic factor in lung with vasoconstrictive and angiogenic properties. Circ Res.

[9] Ghosh S, Singh AK, Aruna B, Mukhopadhyay S, Ehtesham NZ (2003). The genomic organization of mouse resistin reveals major differences from the human resistin: functional implications. Gene.

[10] Fain JN, Cheema PS, Bahouth SW, Lloyd HM (2003). Resistin release by human adipose tissue explants in primary culture. Biochem Biophys Res Commun.

[11] Patel L, Buckels AC, Kinghorn IJ, Murdock PR, Holbrook JD, Plumpton C, Macphee CH, Smith SA (2003). Resistin is expressed in human macrophages and directly regulated by PPAR gamma activators. Biochem Biophys Res Commun.

[12] Deb A, Deshmukh B, Ramteke P, Bhati FK, Bhat MK (2021). Resistin: a journey from metabolism to cancer. Transl Oncol.

[13] Sato H, Muraoka S, Kusunoki N, Masuoka S, Yamada S, Ogasawara H, Imai T, Akasaka Y, Tochigi N, Takahashi H (2017). Resistin upregulates chemokine production by fibroblast-like synoviocytes from patients with rheumatoid arthritis. Arthritis Res Ther.

[14] Yang RZ, Huang Q, Xu A, McLenithan JC, Eisen JA, Shuldiner AR, Alkan S, Gong DW (2003). Comparative studies of resistin expression and phylogenomics in human and mouse. Biochem Biophys Res Commun.

[15] Schinke T, Haberland M, Jamshidi A, Nollau P, Rueger JM, Amling M (2004). Cloning and functional characterization of resistin-like molecule gamma. Biochem Biophys Res Commun.

[16] Banerjee RR, Lazar MA (2001). Dimerization of resistin and resistin-like molecules is determined by a single cysteine. J Biol Chem.

[17] Patel SD, Rajala MW, Rossetti L, Scherer PE, Shapiro L (2004). Disulfide-dependent multimeric assembly of resistin family hormones. Science.

[18] Bing C, Gomez-Ambrosi J, Zabalegui N, Williams G, Trayhurn P (2002). Resistin and RELM-alpha gene expression in white adipose tissue of lactating mice. Biochem Biophys Res Commun.

[19] Rajala MW, Lin Y, Ranalletta M, Yang XM, Qian H, Gingerich R, Barzilai N, Scherer PE (2002). Cell type-specific expression and coregulation of murine resistin and resistin-like molecule-alpha in adipose tissue. Mol Endocrinol.

[20] Lee MR, Lim CJ, Lee YH, Park JG, Sonn SK, Lee MN, Jung IH, Jeong SJ, Jeon S, Lee M (2014). The adipokine retnla modulates cholesterol homeostasis in hyperlipidemic mice. Nat Commun.

[21] Cook PC, Jones LH, Jenkins SJ, Wynn TA, Allen JE, MacDonald AS (2012). Alternatively activated dendritic cells regulate CD4+ T-cell polarization in vitro and in vivo. Proc Natl Acad Sci U S A.

[22] Liu T, Jin H, Ullenbruch M, Hu B, Hashimoto N, Moore B, McKenzie A, Lukacs NW, Phan SH (2004). Regulation of found in inflammatory zone 1 expression in bleomycin-induced lung fibrosis: role of IL-4/IL-13 and mediation via STAT-6. J Immunol.

[23] Raes G, De Baetselier P, Noël W, Beschin A, Brombacher F, Hassanzadeh GhG (2002). Differential expression of FIZZ1 and Ym1 in alternatively versus classically activated macrophages. J Leukoc Biol.

[24] Yamaji-Kegan K, Su Q, Angelini DJ, Myers AC, Cheadle C, Johns RA (2010). Hypoxia-induced mitogenic factor (HIMF/FIZZ1/RELMalpha) increases lung inflammation and activates pulmonary microvascular endothelial cells via an IL-4-dependent mechanism. J Immunol.

[25] Munitz A, Cole ET, Karo-Atar D, Finkelman FD, Rothenberg ME (2012). Resistin-like molecule-α regulates IL-13-induced chemokine production but not allergen-induced airway responses. Am J Respir Cell Mol Biol.

[26] Stütz AM, Pickart LA, Trifilieff A, Baumruker T, Prieschl-Strassmayr E, Woisetschläger M (2003). The Th2 cell cytokines IL-4 and IL-13 regulate found in inflammatory zone 1/resistin-like molecule alpha gene expression by a STAT6 and CCAAT/enhancer-binding protein-dependent mechanism. J Immunol.

[27] Karo-Atar D, Moshkovits I, Eickelberg O, Königshoff M, Munitz A (2013). Paired immunoglobulin-like receptor-B inhibits pulmonary fibrosis by suppressing profibrogenic properties of alveolar macrophages. Am J Respir Cell Mol Biol.

[28] Renigunta A, Hild C, Rose F, Klepetko W, Grimminger F, Seeger W, Hänze J (2006). Human RELMbeta is a mitogenic factor in lung cells and induced in hypoxia. FEBS Lett.

[29] Shojima N, Ogihara T, Inukai K, Fujishiro M, Sakoda H, Kushiyama A, Katagiri H, Anai M, Ono H, Fukushima Y (2005). Serum concentrations of resistin-like molecules beta and gamma are elevated in high-fat-fed and obese db/db mice, with increased production in the intestinal tract and bone marrow. Diabetologia.

[30] Herbert DR, Yang JQ, Hogan SP, Groschwitz K, Khodoun M, Munitz A, Orekov T, Perkins C, Wang Q, Brombacher F (2009). Intestinal epithelial cell secretion of RELM-beta protects against gastrointestinal worm infection. J Exp Med.

[31] Jiang Y, Zhou X, Hu R, Dai A (2018). TGF-β1-induced SMAD2/3/4 activation promotes RELM-β transcription to modulate the endothelium-mesenchymal transition in human endothelial cells. Int J Biochem Cell Biol.

[32] Wang ML, Shin ME, Knight PA, Artis D, Silberg DG, Suh E, Wu GD (2005). Regulation of RELM/FIZZ isoform expression by Cdx2 in response to innate and adaptive immune stimulation in the intestine. Am J Physiol Gastrointest Liver Physiol.

[33] Fujio J, Kushiyama A, Sakoda H, Fujishiro M, Ogihara T, Fukushima Y, Anai M, Horike N, Kamata H, Uchijima Y (2008). Regulation of gut-derived resistin-like molecule beta expression by nutrients. Diabetes Res Clin Pract.

[34] Bhatia S, Prabhu PN, Benefiel AC, Miller MJ, Chow J, Davis SR, Gaskins HR (2015). Galacto-oligosaccharides may directly enhance intestinal barrier function through the modulation of goblet cells. Mol Nutr Food Res.

[35] Pinton P, Graziani F, Pujol A, Nicoletti C, Paris O, Ernouf P, Di Pasquale E, Perrier J, Oswald IP, Maresca M (2015). Deoxynivalenol inhibits the expression by goblet cells of intestinal mucins through a PKR and MAP kinase dependent repression of the resistin-like molecule β. Mol Nutr Food Res.

[36] Yamaji-Kegan K, Su Q, Angelini DJ, Champion HC, Johns RA (2006). Hypoxia-induced mitogenic factor has proangiogenic and proinflammatory effects in the lung via VEGF and VEGF receptor-2. Am J Physiol Lung Cell Mol Physiol.

[37] Johns RA, Takimoto E, Meuchel LW, Elsaigh E, Zhang A, Heller NM, Semenza GL, Yamaji-Kegan K (2016). Hypoxia-inducible factor 1α is a critical downstream mediator for hypoxia-induced mitogenic factor (FIZZ1/RELMα)-induced pulmonary hypertension. Arterioscler Thromb Vasc Biol.

[38] Fan C, Meuchel LW, Su Q, Angelini DJ, Zhang A, Cheadle C, Kolosova I, Makarevich OD, Yamaji-Kegan K, Rothenberg ME (2015). Resistin-like molecule *α* in allergen-induced pulmonary vascular remodeling. Am J Respir Cell Mol Biol.

[39] Lin Q, Fan C, Skinner JT, Hunter EN, Macdonald AA, Illei PB, Yamaji-Kegan K, Johns RA (2019). RELMα licenses macrophages for damage-associated molecular pattern activation to instigate pulmonary vascular remodeling. J Immunol.

[40] Wang WY, Chen Y, Su X, Tang D, Ben QW, Yao WY, Chen P, Yuan YZ (2016). Resistin-like molecule-α causes lung injury in rats with acute pancreatitis by activating the PI-3K/Akt-NF-κB pathway and promoting inflammatory cytokine release. Curr Mol Med.

[41] Nair MG, Du Y, Perrigoue JG, Zaph C, Taylor JJ, Goldschmidt M, Swain GP, Yancopoulos GD, Valenzuela DM, Murphy A (2009). Alternatively activated macrophage-derived RELM-{alpha} is a negative regulator of type 2 inflammation in the lung. J Exp Med.

[42] Chen F, Wu W, Jin L, Millman A, Palma M, El-Naccache DW, Lothstein KE, Dong C, Edelblum KL, Gause WC (2018). B cells produce the tissue-protective protein RELMα during helminth infection, which inhibits IL-17 expression and limits emphysema. Cell Rep.

[43] Munitz A, Waddell A, Seidu L, Cole ET, Ahrens R, Hogan SP, Rothenberg ME (2008). Resistin-like molecule alpha enhances myeloid cell activation and promotes colitis. J Allergy Clin Immunol.

[44] Munitz A, Seidu L, Cole ET, Ahrens R, Hogan SP, Rothenberg ME (2009). Resistin-like molecule alpha decreases glucose tolerance during intestinal inflammation. J Immunol.

[45] Osborne LC, Joyce KL, Alenghat T, Sonnenberg GF, Giacomin PR, Du Y, Bergstrom KS, Vallance BA, Nair MG (2013). Resistin-like molecule α promotes pathogenic Th17 cell responses and bacterial-induced intestinal inflammation. J Immunol.

[46] Chen G, Chan AJ, Chung JI, Jang JC, Osborne LC, Nair MG (2014). Polarizing the T helper 17 response in *Citrobacter rodentium* infection via expression of resistin-like molecule α. Gut Microbes.

[47] Hogan SP, Seidu L, Blanchard C, Groschwitz K, Mishra A, Karow ML, Ahrens R, Artis D, Murphy AJ, Valenzuela DM (2006). Resistin-like molecule beta regulates innate colonic function: barrier integrity and inflammation susceptibility. J Allergy Clin Immunol.

[48] McVay LD, Keilbaugh SA, Wong TM, Kierstein S, Shin ME, Lehrke M, Lefterova MI, Shifflett DE, Barnes SL, Cominelli F (2006). Absence of bacterially induced RELMbeta reduces injury in the dextran sodium sulfate model of colitis. J Clin Invest.

[49] Morampudi V, Dalwadi U, Bhinder G, Sham HP, Gill SK, Chan J, Bergstrom KS, Huang T, Ma C, Jacobson K (2016). The goblet cell-derived mediator RELM-β drives spontaneous colitis in Muc2-deficient mice by promoting commensal microbial dysbiosis. Mucosal Immunol.

[50] Barnes SL, Vidrich A, Wang ML, Wu GD, Cominelli F, Rivera-Nieves J, Bamias G, Cohn SM (2007). Resistin-like molecule beta (RELMbeta/FIZZ2) is highly expressed in the ileum of SAMP1/YitFc mice and is associated with initiation of ileitis. J Immunol.

[51] Nair MG, Guild KJ, Du Y, Zaph C, Yancopoulos GD, Valenzuela DM, Murphy A, Stevens S, Karow M, Artis D (2008). Goblet cell-derived resistin-like molecule beta augments CD4+ T cell production of IFN-gamma and infection-induced intestinal inflammation. J Immunol.

[52] Che L, Yu C, Chen G, Lin J, Xie Z, Xia T, Luo W, Cai X, Liu S (2021). The inflammatory response induced by RELMβ upregulates IL-8 and IL-1β expression in bronchial epithelial cells in COPD. Int J Chron Obstruct Pulmon Dis.

[53] Lin Q, Fan C, Gomez-Arroyo J, Van Raemdonck K, Meuchel LW, Skinner JT, Everett AD, Fang X, Macdonald AA, Yamaji-Kegan K (2019). HIMF (hypoxia-induced mitogenic factor) signaling mediates the HMGB1 (high mobility group box 1)-dependent endothelial and smooth muscle cell crosstalk in pulmonary hypertension. Arterioscler Thromb Vasc Biol.

[54] Angelini DJ, Su Q, Kolosova IA, Fan C, Skinner JT, Yamaji-Kegan K, Collector M, Sharkis SJ, Johns RA (2010). Hypoxia-induced mitogenic factor (HIMF/FIZZ1/RELM alpha) recruits bone marrow-derived cells to the murine pulmonary vasculature. PLoS ONE.

[55] Kolosova IA, Angelini D, Fan C, Skinner J, Cheadle C, Johns RA (2013). Resistin-like molecule α stimulates proliferation of mesenchymal stem cells while maintaining their multipotency. Stem Cells Dev.

[56] Mavi P, Niranjan R, Dutt P, Zaidi A, Shukla JS, Korfhagen T, Mishra A (2014). Allergen-induced resistin-like molecule-α promotes esophageal epithelial cell hyperplasia in eosinophilic esophagitis. Am J Physiol Gastrointest Liver Physiol.

[57] Tian H, Liu L, Wu Y, Wang R, Jiang Y, Hu R, Zhu L, Li L, Fang Y, Yang C (2021). Resistin-like molecule β acts as a mitogenic factor in hypoxic pulmonary hypertension via the Ca^2+^-dependent PI3K/Akt/mTOR and PKC/MAPK signaling pathways. Respir Res.

[58] Han L, Song N, Hu X, Zhu A, Wei X, Liu J, Yuan S, Mao W, Chen X (2020). Inhibition of RELM-β prevents hypoxia-induced overproliferation of human pulmonary artery smooth muscle cells by reversing PLC-mediated KCNK3 decline. Life Sci.

[59] Lin C, Li X, Luo Q, Yang H, Li L, Zhou Q, Li Y, Tang H, Wu L (2017). RELM-β promotes human pulmonary artery smooth muscle cell proliferation via FAK-stimulated surviving. Exp Cell Res.

[60] Fang C, Meng Q, Wu H, Eid G, Zhang G, Zhang X, Yang S, Huang K, Lee TH, Corrigan CJ (2012). Resistin-like molecule-β is a human airway remodelling mediator. Eur Respir J.

[61] Fang CL, Yin LJ, Sharma S, Kierstein S, Wu HF, Eid G, Haczku A, Corrigan CJ, Ying S (2015). Resistin-like molecule-β (RELM-β) targets airways fibroblasts to effect remodelling in asthma: from mouse to man. Clin Exp Allergy.

[62] Wang Y, Zhang Y, Gao X, Qian J, Yang J, Sun W, Wang H, Yang Y (2021). Resistin-like molecule beta augments phenotypic modulation of human aortic smooth muscle cell triggered by high glucose. Endocr J.

[63] Bergstrom KS, Morampudi V, Chan JM, Bhinder G, Lau J, Yang H, Ma C, Huang T, Ryz N, Sham HP (2015). Goblet cell derived RELM-β recruits CD4+ t cells during infectious colitis to promote protective intestinal epithelial cell proliferation. PLoS Pathog.

[64] Kumamoto Y, Camporez JPG, Jurczak MJ, Shanabrough M, Horvath T, Shulman GI, Iwasaki A (2016). CD301b(+) mononuclear phagocytes maintain positive energy balance through secretion of resistin-like molecule alpha. Immunity.

[65] Kushiyama A, Shojima N, Ogihara T, Inukai K, Sakoda H, Fujishiro M, Fukushima Y, Anai M, Ono H, Horike N (2005). Resistin-like molecule beta activates MAPKs, suppresses insulin signaling in hepatocytes, and induces diabetes, hyperlipidemia, and fatty liver in transgenic mice on a high fat diet. J Biol Chem.

[66] Rajala MW, Obici S, Scherer PE, Rossetti L (2003). Adipose-derived resistin and gut-derived resistin-like molecule-beta selectively impair insulin action on glucose production. J Clin Invest.

[67] Krimi RB, Letteron P, Chedid P, Nazaret C, Ducroc R, Marie JC (2009). Resistin-like molecule-beta inhibits SGLT-1 activity and enhances GLUT2-dependent jejunal glucose transport. Diabetes.

[68] Harris TA, Gattu S, Propheter DC, Kuang Z, Bel S, Ruhn KA, Chara AL, Edwards M, Zhang C, Jo JH (2019). Resistin-like molecule α provides vitamin-A-dependent antimicrobial protection in the skin. Cell Host Microbe.

[69] Specian RD, Oliver MG (1991). Functional biology of intestinal goblet cells. Am J Physiol.

[70] Krimi RB, Kotelevets L, Dubuquoy L, Plaisancié P, Walker F, Lehy T, Desreumaux P, Van Seuningen I, Chastre E, Forgue-Lafitte ME (2008). Resistin-like molecule beta regulates intestinal mucous secretion and curtails TNBS-induced colitis in mice. Inflamm Bowel Dis.

[71] He W, Wang ML, Jiang HQ, Steppan CM, Shin ME, Thurnheer MC, Cebra JJ, Lazar MA, Wu GD (2003). Bacterial colonization leads to the colonic secretion of RELMbeta/FIZZ2, a novel goblet cell-specific protein. Gastroenterology.

[72] Humbert M, Guignabert C, Bonnet S, Dorfmüller P, Klinger JR, Nicolls MR, Olschewski AJ, Pullamsetti SS, Schermuly RT, Stenmark KR (2019). Pathology and pathobiology of pulmonary hypertension: state of the art and research perspectives. Eur Respir J.

[73] Zhang L, Wang M, Kang X, Boontheung P, Li N, Nel AE, Loo JA (2009). Oxidative stress and asthma: proteome analysis of chitinase-like proteins and FIZZ1 in lung tissue and bronchoalveolar lavage fluid. J Proteome Res.

[74] Tao B, Kumar S, Gomez-Arroyo J, Fan C, Zhang A, Skinner J, Hunter E, Yamaji-Kegan K, Samad I, Hillel AT (2021). Resistin-like molecule α dysregulates cardiac bioenergetics in neonatal rat cardiomyocytes. Front Cardiovasc Med.

[75] Angelini DJ, Su Q, Yamaji-Kegan K, Fan C, Teng X, Hassoun PM, Yang SC, Champion HC, Tuder RM, Johns RA (2009). Resistin-like molecule-beta in scleroderma-associated pulmonary hypertension. Am J Respir Cell Mol Biol.

[76] Dong L, Wang SJ, Camoretti-Mercado B, Li HJ, Chen M, Bi WX (2008). FIZZ1 plays a crucial role in early stage airway remodeling of OVA-induced asthma. J Asthma.

[77] Liu T, Dhanasekaran SM, Jin H, Hu B, Tomlins SA, Chinnaiyan AM, Phan SH (2004). FIZZ1 stimulation of myofibroblast differentiation. Am J Pathol.

[78] Lee MR, Shim D, Yoon J, Jang HS, Oh SW, Suh SH, Choi JH, Oh GT (2014). Retnla overexpression attenuates allergic inflammation of the airway. PLoS ONE.

[79] Mishra A, Wang M, Schlotman J, Nikolaidis NM, DeBrosse CW, Karow ML, Rothenberg ME (2007). Resistin-like molecule-beta is an allergen-induced cytokine with inflammatory and remodeling activity in the murine lung. Am J Physiol Lung Cell Mol Physiol.

[80] Grainge C, Dulay V, Ward J, Sammut D, Davies E, Green B, Lau L, Cottey L, Haitchi HM, Davies DE (2012). Resistin-like molecule-β is induced following bronchoconstriction of asthmatic airways. Respirology.

[81] LeMessurier KS, Palipane M, Tiwary M, Gavin B, Samarasinghe AE (2018). Chronic features of allergic asthma are enhanced in the absence of resistin-like molecule-beta. Sci Rep.

[82] Choi JY, Hur J, Jeon S, Jung CK, Rhee CK (2022). Effects of human adipose tissue- and bone marrow-derived mesenchymal stem cells on airway inflammation and remodeling in a murine model of chronic asthma. Sci Rep.

[83] Burgess JK, Mauad T, Tjin G, Karlsson JC, Westergren-Thorsson G (2016). The extracellular matrix - the under-recognized element in lung disease?. J Pathol.

[84] Ho L, Yip A, Lao F, Botelho F, Richards CD (2020). RELMα is induced in airway epithelial cells by oncostatin M without requirement of STAT6 or IL-6 in mouse lungs in vivo. Cells.

[85] Doherty TA, Khorram N, Sugimoto K, Sheppard D, Rosenthal P, Cho JY, Pham A, Miller M, Croft M, Broide DH (2012). Alternaria induces STAT6-dependent acute airway eosinophilia and epithelial FIZZ1 expression that promotes airway fibrosis and epithelial thickness. J Immunol.

[86] Liu T, Baek HA, Yu H, Lee HJ, Park BH, Ullenbruch M, Liu J, Nakashima T, Choi YY, Wu GD (2011). FIZZ2/RELM-β induction and role in pulmonary fibrosis. J Immunol.

[87] Chen G, Wang SH, Jang JC, Odegaard JI, Nair MG (2016). Comparison of RELMα and RELMβ single- and double-gene-deficient mice reveals that RELMα expression dictates inflammation and worm expulsion in Hookworm infection. Infect Immun.

[88] Nair MG, Gallagher IJ, Taylor MD, Loke P, Coulson PS, Wilson RA, Maizels RM, Allen JE (2005). Chitinase and fizz family members are a generalized feature of nematode infection with selective upregulation of Ym1 and Fizz1 by antigen-presenting cells. Infect Immun.

[89] Batugedara HM, Li J, Chen G, Lu D, Patel JJ, Jang JC, Radecki KC, Burr AC, Lo DD, Dillman AR (2018). Hematopoietic cell-derived RELMα regulates hookworm immunity through effects on macrophages. J Leukoc Biol.

[90] Krljanac B, Schubart C, Naumann R, Wirtz S, Culemann S, Krönke G, Voehringer D (2019). RELMα-expressing macrophages protect against fatal lung damage and reduce parasite burden during helminth infection. Sci Immunol.

[91] Pesce JT, Ramalingam TR, Wilson MS, Mentink-Kane MM, Thompson RW, Cheever AW, Urban JF, Wynn TA (2009). Retnla (relmalpha/fizz1) suppresses helminth-induced Th2-type immunity. PLoS Pathog.

[92] Soga K, Yamada M, Naito Y, Yoshikawa T, Arizono N (2013). Mucin-related molecular responses of bronchial epithelial cells in rats infected with the nematode nippostrongylus Brasiliensis. ISRN Parasitol.

[93] Sun R, Urban JF, Notari L, Vanuytsel T, Madden KB, Bohl JA, Ramalingam TR, Wynn TA, Zhao A, Shea-Donohue T (2016). Interleukin-13 receptor α1-dependent responses in the intestine are critical to parasite clearance. Infect Immun.

[94] Hung LY, Lewkowich IP, Dawson LA, Downey J, Yang Y, Smith DE, Herbert DR (2013). IL-33 drives biphasic IL-13 production for noncanonical type 2 immunity against hookworms. Proc Natl Acad Sci U S A.

[95] Artis D, Wang ML, Keilbaugh SA, He W, Brenes M, Swain GP, Knight PA, Donaldson DD, Lazar MA, Miller HR (2004). RELMbeta/FIZZ2 is a goblet cell-specific immune-effector molecule in the gastrointestinal tract. Proc Natl Acad Sci U S A.

[96] Chen L, Luo Q, Shang Y, He X, Xu Y, Gao Z (2021). Predictive and prognostic utility of the serum level of resistin-like molecule beta for risk stratification in patients with community-acquired pneumonia. Pathogens.

[97] Watanabe K, Itoh K, Park SH, Kaku M, Ishii K, Sasano H, Naitoh T, Unno M, Fukushima K (2020). Resistin-like molecule beta, a colonic epithelial protein, exhibits antimicrobial activity against Staphylococcus aureus including methicillin-resistant strains. Surg Today.

[98] Propheter DC, Chara AL, Harris TA, Ruhn KA, Hooper LV (2017). Resistin-like molecule β is a bactericidal protein that promotes spatial segregation of the microbiota and the colonic epithelium. Proc Natl Acad Sci U S A.

[99] Ren C, Dokter-Fokkens J, Figueroa Lozano S, Zhang Q, de Haan BJ, Zhang H, Faas MM, de Vos P (2019). Fibroblasts impact goblet cell responses to lactic acid bacteria after exposure to inflammatory cytokines and mucus disruptors. Mol Nutr Food Res.

[100] Ren C, Dokter-Fokkens J, Figueroa Lozano S, Zhang Q, de Haan BJ, Zhang H, Faas MM, de Vos P (2018). Lactic acid bacteria may impact intestinal barrier function by modulating goblet cells. Mol Nutr Food Res.

[101] Choudhry N, Scott F, Edgar M, Sanger GJ, Kelly P (2021). Reversal of pathogen-induced barrier defects in intestinal epithelial cells by contra-pathogenicity agents. Dig Dis Sci.

[102] Zhang HM, Li XY, He ZY, Xu LZ, Jin Q, Tan H (2013). Resistin-like molecule alpha enhances the proliferation and migration of aortic vascular smooth muscle cells. Cardiology.

[103] Kushiyama A, Sakoda H, Oue N, Okubo M, Nakatsu Y, Ono H, Fukushima T, Kamata H, Nishimura F, Kikuchi T (2013). Resistin-like molecule β is abundantly expressed in foam cells and is involved in atherosclerosis development. Arterioscler Thromb Vasc Biol.

[104] Lilleker J, Murphy S, Cooper R (2016). Selected aspects of the current management of myositis. Ther Adv Musculoskelet Dis.

[105] Gono T, Kawaguchi Y, Sugiura T, Ichida H, Takagi K, Katsumata Y, Hanaoka M, Okamoto Y, Ota Y, Yamanaka H (2010). Interleukin-18 is a key mediator in dermatomyositis: potential contribution to development of interstitial lung disease. Rheumatology (Oxford).

[106] Su CM, Wang IC, Liu SC, Sun Y, Jin L, Wang SW, Lee HP, Tseng WP, Tang CH (2017). Hypoxia induced mitogenic factor (HIMF) triggers angiogenesis by increasing interleukin-18 production in myoblasts. Sci Rep.

[107] Meng X, Zhang K, Kong J, Xu L, An G, Qin W, Li J, Zhang Y (2017). Deletion of resistin-like molecule-beta attenuates angiotensin II-induced abdominal aortic aneurysm. Oncotarget.

[108] Chen P, Zhao D, Wang W, Zhang Y, Yuan Y, Wang L, Wu Y (2015). High expression of RELM-α correlates with poor prognosis and promotes angiogenesis in gastric cancer. Oncol Rep.

[109] Zheng L, Weng M, He J, Yang X, Jiang G, Tong Q (2010). Expression of resistin-like molecule beta in gastric cancer: its relationship with clinicopathological parameters and prognosis. Virchows Arch.

[110] Jiang R, Zhao C, Wang X, Wang S, Sun X, Tian Y, Song W (2016). Resistin-like molecule-β promotes invasion and migration of gastric carcinoma cells. Med Sci Monit.

[111] Correa P (1988). Chronic gastritis: a clinico-pathological classification. Am J Gastroenterol.

[112] Toller IM, Altmeyer M, Kohler E, Hottiger MO, Müller A (2010). Inhibition of ADP ribosylation prevents and cures helicobacter-induced gastric preneoplasia. Cancer Res.

[113] Li HJ, Fang EH, Wang JQ, Zheng LD, Tong QS (2020). Helicobacter pylori Infection facilitates the expression of resistin-like molecule beta in gastric carcinoma and precursor lesions. Curr Med Sci.

[114] Provenzale D, Kemp JA, Arora S, Wong JB (1994). A guide for surveillance of patients with Barrett's esophagus. Am J Gastroenterol.

[115] DeMeester SR, DeMeester TR (2000). Columnar mucosa and intestinal metaplasia of the esophagus: fifty years of controversy. Ann Surg.

[116] Zheng L, Tong Q, Weng M, He J, Lv Q, Wu Z, Du Z, Mei H, Hou X (2010). Expression of resistin-like molecule beta in Barrett's esophagus: a novel biomarker for metaplastic epithelium. Dig Dis Sci.

[117] Zheng LD, Tong QS, Weng MX, He J, Lv Q, Pu JR, Jiang GS, Cai JB, Liu Y, Hou XH (2009). Enhanced expression of resistin-like molecule beta in human colon cancer and its clinical significance. Dig Dis Sci.

[118] Neilson AP, Djuric Z, Land S, Kato I (2011). Plasma levels of resistin-like molecule beta in humans. Cancer Epidemiol.

[119] Al-Azzawi HH, Mathur A, Lu D, Swartz-Basile DA, Nakeeb A, Pitt HA (2007). Resistin-like molecule alpha reduces gallbladder optimal tension. J Gastrointest Surg.

[120] Okubo H, Kushiyama A, Sakoda H, Nakatsu Y, Iizuka M, Taki N, Fujishiro M, Fukushima T, Kamata H, Nagamachi A (2016). Involvement of resistin-like molecule β in the development of methionine-choline deficient diet-induced non-alcoholic steatohepatitis in mice. Sci Rep.

[121] Hosoya A, Takahama A, Nakamura H (2017). Localization of RELM-β/FIZZ2 Is associated with cementum formation. Anat Rec (Hoboken).

